# Photochemical Catalyst-Free Synthesis of Pyrrolidines via a Hofmann–Loffler–Freytag Reaction

**DOI:** 10.3390/molecules31111963

**Published:** 2026-06-05

**Authors:** Athina S. J. Shkembi, Luca Pasqualon, Stamatis K. Serviou, Manos V. G. Lantzanakis, Christoforos G. Kokotos

**Affiliations:** Laboratory of Organic Chemistry, Department of Chemistry, National and Kapodistrian University of Athens, 15772 Athens, Greece; athinashk@chem.uoa.gr (A.S.J.S.); luca.pasqualon@studio.unibo.it (L.P.); stamserv@chem.uoa.gr (S.K.S.); manoslan@chem.uoa.gr (M.V.G.L.)

**Keywords:** photochemistry, Hofmann–Loffler cyclization, catalyst-free, pyrrolidines

## Abstract

The Hoffman–Loffler–Freytag (HLF) reaction is one of the first transformations to achieve remote C(sp^3^)-H functionalization and simultaneously provide useful building blocks from readily available reagents. Herein, we propose a photochemical protocol for the HLF cyclization, utilizing UVA light as the initiating force under catalyst-free conditions, followed by base-mediated cyclization, in order to synthesize pyrrolidines in a mild and sustainable manner.

## 1. Introduction

Heterocyclic structures have emerged as key contributing scaffolds in drug discovery, with the pyrrolidine moiety being ubiquitously detected in a variety of pharmacologically and biologically active compounds [1]. Pyrrolidine derivatives exhibit notable antibacterial and anti-inflammatory properties and are continuously utilized as templates for potential drug candidates [2]. Therefore, the establishment of an efficient and sustainable strategy for the construction of pyrrolidine rings would provide a powerful synthetic tool for chemists, both in academia and industry.

The Hofmann–Löffler–Freytag reaction (HLF) was first reported by Hofmann in the late 1800s [3]; however, it was not recognized until a few decades later, when Löffler and Freytag expanded his work, demonstrating the generality of employing halogenated amines towards the synthesis of pyrrolidine rings and being one of the first examples of remote C(sp^3^)-H functionalization [4]. Remarkably, the reaction mechanism was elucidated almost 50 years later, when Wawzonek and Thelen [5], as well as Corey and Hertler [6], uncovered the radical nature of this transformation. In recent years, modern methods for radical generation have been implemented in the development of a variety of HLF protocols, utilizing metal catalysis, under milder and more practical conditions, compared to the original harsh protocols [7,8].

**Scheme 1:**
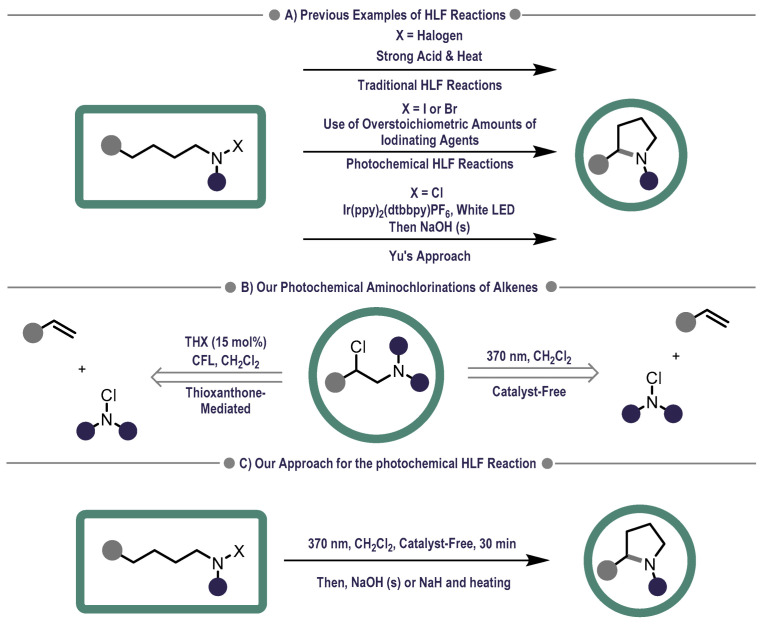
Selected reports in the literature and this work.

Light-induced activation offers a modern and inherently green strategy for generating reactive radical intermediates. Photochemistry has witnessed a renaissance since the start of this century, unlocking chemical transformations that were previously beyond reach [9,10,11,12]. The HLF reaction has also been heavily impacted by this revival, leading to a variety of protocols utilizing visible light to achieve the synthesis of pyrrolidines (Scheme 1A). In 2015, Herrera adapted a non-photochemical protocol, reported by Fan in 2007 [13], employing photochemical conditions to produce pyrrolidines and pyrrolidones from iodinated amines [14]. In the same year, Muñiz and Martínez utilized a similar catalytic system that did not require heating to produce the desired pyrrolidines [15]. Subsequently, Muñiz and his co-workers published a series of protocols producing iodoamides, using iodine catalysis or iodinating agents, such as *N*-iodosuccinimide, and utilizing them to produce pyrrolidines via a HLF cyclization [16,17,18]. In 2016, Nagib introduced a novel triiodide-mediated photochemical method that led to the production of pyrrolidine derivatives in an efficient method [19]. Five years later, Moriyama used a hypervalent iodine reagent to perform this cyclization under fluorescent light [20]. All these methodologies heavily rely on the weak iodine–nitrogen bond, which undergoes facile cleavage under irradiation. However, the in situ generation of the unstable iodoamides usually requires overstoichiometric amounts of reagents. On the other hand, Yu introduced a different approach to the photochemical HLF cyclization, using the much more stable chloroamines and Ir(ppy)_2_(dtbbpy)PF_6_ to homolytically cleave the nitrogen chlorine bond and with the use of an external base to cyclize the produced *δ*-chloroamines to the corresponding pyrrolidines (Scheme 1A) [21]. More recently, Lu and his co-workers combined photocatalysis and organocatalysis to introduce an asymmetric version of the HLF reaction via a carbenium ion complex [22].

The Kokotos’ group has experience in the development of photochemical protocols [23,24,25]; among which, a catalyst-free [24] and a thioxanthone-mediated protocol [25] for the homolytic cleavage of the N-Cl bond of *N*-chloroamides, enabling the aminochlorination of double bonds, were introduced (Scheme 1B). Combining our knowledge on these protocols with the previously mentioned literature, we herein provide a protocol for the UVA-promoted catalyst-free remote C(sp^3^)-H bond functionalization via an HLF cyclization to afford pyrrolidines in a mild and sustainable manner (Scheme 1C).

## 2. Results and Discussion

Drawing inspiration from Yu’s approach and our previously reported catalyst-free aminochlorination of alkenes, we initiated an optimization study for a photochemical HLF reaction utilizing N-chloroamide **1a** (Table 1). We began by examining the optimum wavelength for this reaction to take place and during this process, we discovered that in only 30 min under irradiation from a 370 nm Kessil lamp, pyrrolidine **2a** was isolated in 56% yield (Table 1, entry 1). Interestingly, irradiation by a second generation 370 nm LED lamp led to a significantly reduced yield, affording mostly non-chlorinated or non-cyclized by-products (Table 1, entry 2). A close second was the 400 nm lamp that provided the desired product in 41% isolated yield (Table 1, entry 4). Meanwhile, all other wavelengths (390 nm, 427 nm, 440 nm, 456 nm, 467 nm, 525 nm or 640 nm (Table 1, entries 3 and 5–10) available to us were not suitable, with the higher wavelength irradiation providing an ineffective environment for the nitrogen–chlorine bond cleavage.

Our next objective was to identify the most suitable solvent for this transformation (Table 2). Although acetonitrile afforded the highest isolated yield of the cyclized product **2a** (54%, Table 2, entry 1), reactions conducted in CH_2_Cl_2_ afforded significantly higher combined yields of the δ-chloroamine intermediate **3a** and the cyclized product **2a** (Table 2, entry 2). This suggested that the overall formation of these two species was a more meaningful metric than the isolated yield of the cyclized product alone, since the appropriate choice of base could subsequently direct the reaction toward the desired cyclization pathway. Among the other solvents, only EtOAc and MeOH provided substantial results (Table 2, entries 3–9); however, none of them outperformed CH_2_Cl_2_. Based on this, we selected CH_2_Cl_2_ for further optimization and proceeded to evaluate the most suitable base for the transformation.

Next, we studied the influence of the base in the photochemical step (Table 3). Organic bases, such as pyridine or Et_3_N, proved ineffective (Table 3, entries 7 and 8), whereas inorganic bases K_2_CO_3_ and Na_2_CO_3_ delivered the highest yields (Table 3, entries 3 and 6), with K_2_CO_3_ emerging as the optimum choice, affording a 76% isolated yield. Notably, omitting the base during the photochemical step led to a sharp decrease in yield (Table 3, entry 9). In addition, switching the solvent back to MeCN confirmed that our interpretation regarding the importance of the δ-chlorinated intermediate was proven valid, as the yield dropped to 68%, while the increase in the equivalents of NaOH from 2.5 to 3.5 in the following step led to a significant decrease in the yield of **2a** (Table 3, entries 4 and 5).

With the optimum reaction conditions in hand, we proceeded by testing various substrates for the HLF cyclization. However, their application to the standard conditions led to drastically diminished yields and the predominant formation of the δ-chlorinated intermediate, instead of the cyclized one. We therefore evaluated a range of bases using *N*-chloro-*N*-dodecyl-4-methylbenzenesylfonamide (**1b**), which failed to cyclize under the standard conditions (Table 4, entry 1). By using DBU at different equivalents and at varying temperatures (Table 4, entries 2–6), the most satisfactory results were observed with 7.5 equivalents and heating at 70 °C for 40 h (83% yield, Table 4, entry 6). In order to improve the sustainability of our protocol, further optimization studies were conducted, which revealed that Et_3_N was ineffective in promoting the cyclization (Table 4, entry 7). Meanwhile, NaH emerged as the optimum base at 4.0 equivalents, affording the product at 95% conversion in only 18 h at 100 °C (Table 4, entry 11). Attempts to lower the reaction temperature did not prove fruitful (Table 4, entries 9 and 10) and, hence, heating at 100 °C was deemed essential for the final cyclization of substrates **1b**–**i**.

**Scheme 2:**
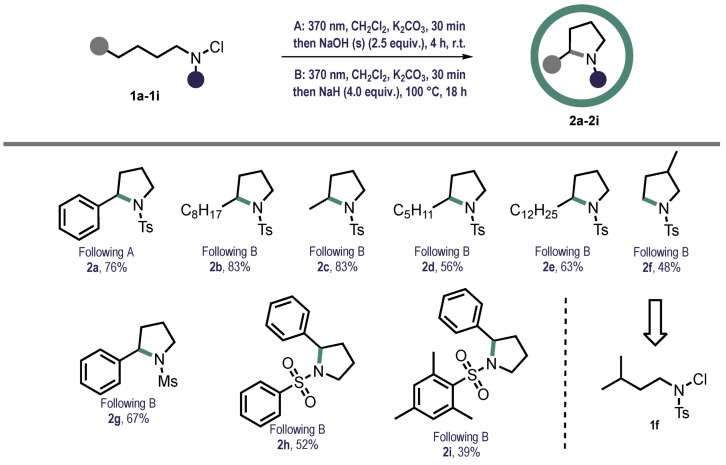
Substrate scope.

Ultimately, these studies established a practical protocol that enables efficient cyclization of chloroamide **1a** under mild conditions and, with an adjustment of the base and temperature, provides access to pyrrolidines from the remaining chloroamide substrates. Now with both procedures established, we examined the scope of chloroamides (Scheme 2). We first varied the carbon chain on the chloroamide from a phenyl group to an aliphatic chain with five, nine, twelve or sixteen carbon atoms (Scheme 2, **2b**–**e**), achieving remote C-H functionalization four carbons away from the nitrogen atom. Furthermore, as expected, using an alkyl chain forming a primary radical resulted in lower efficiency, affording the product in only 48% yield (Scheme 2, **2f**). Ultimately, we investigated the effect of the *N*-sulfonyl substituent. In addition to the tosylamide moiety, the mesyl and phenyl sulfonyl derivatives proved to be suitable substrates, affording the desired products in satisfactory yields (Scheme 2, **2g** and **2h**). In contrast, the mesityl group was substantially less reactive, affording the desired product in 39% yield (Scheme 2, **2i**).

**Scheme 3:**
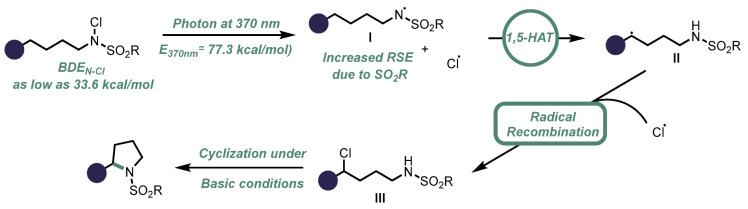
Plausible reaction mechanism.

Mechanistically, this transformation is well-established in the literature and takes place via an energy transfer process [24] or a direct homolytic cleavage of the weak nitrogen–halogen bond [25]. This leads to the generation of the nitrogen-centered radical **I** (Scheme 3) and is followed by a 1,5-HAT event that is highly favorable compared to other intramolecular HAT events, as reported by Šakić and co-workers [26]. Eventually, the generated C-centered radical **II,** through a radical recombination process, affords intermediate δ-chloroamide **III**. In our system, homolytic cleavage of the N-Cl bond can be achieved in the absence of an external catalyst, due to the direct irradiation of the reaction mixture. The high energy photons provided from a 370 nm LED (≃77.3 kcal/mol, based on the Planck-Einstein equation) are of sufficient enough energy to cleave the N-Cl bond of a chlorosulfonylamide, whose reports of BDEs have been reported to be as low as 36.4 kcal/mol [27]. Additionally, the high radical stabilization energy of sulfonyl-substituted amines further improves this process’s efficiency towards the desired transition states [26]. Finally, under substrate-dependent basic conditions, this intermediate cyclizes to furnish the desired pyrrolidine.

## 3. Materials and Methods

### 3.1. General Remarks

Chromatographic purification was accomplished using forced-flow chromatography. Thin-layer chromatography (TLC) was performed on aluminum backed silica plates. Visualization of the developed chromatogram was performed by fluorescence quenching, using phosphomolybdic acid, ninhydrin or vanillin stains. Mass spectra (ESI) were recorded on a LC-MS spectrometer (Thermo Fisher Scientific, Waltham, MA, USA). HRMS spectra were recorded on a QTOF spectrometer (Bruker Daltonics, Bremen, Germany). ^1^H- and ^13^C-NMR spectra were recorded on a Bruker^®^ Avance NEO (Bruker, Fällanden, Switzerland) (400 MHz and 100 MHz, respectively) spectrometer in CDCl_3_ and are internally referenced to residual solvent signals. Data for ^1^H-NMR are reported as follows: chemical shift (δ ppm), integration, multiplicity (s = singlet, d = doublet, t = triplet, q = quartet, m = multiplet, br s = broad signal), coupling constant and assignment. Data for ^13^C-NMR are reported in terms of chemical shift (δ ppm). Kessil lamps PR160L (Kessil, Richmond, CA, USA) were used as the irradiation source. For the experiments, the intensity of the Kessil lamps was controlled at the maximum level with power consumption: 370 nm (max 43 W), 390 nm (max 52 W), 400 nm, 427 nm and 440 nm (max 45 W), 456 nm (max 50 W), 467 nm (max 44 W) and 525 nm (max 44 W). The photochemical reactor used is a 3D-printed model introduced by the Nöel research group [28].

### 3.2. General Procedure A for the Photochemical HLF Cyclization

In a glass vial, *N*-chlorosulfonamide (0.20 mmol, 1.0 equiv.), K_2_CO_3_ (0.24 mmol, 1.2 equiv.) and CH_2_Cl_2_ (4.0 mL) were added and the vial was sealed. The reaction mixture was degassed by bubbling with argon for 5 min. The reaction mixture was left stirring under UVA LED irradiation (370 nm) and an argon atmosphere for 30 min. Afterwards, solid NaOH was added and the reaction mixture was left stirring for an additional 4 h. After reaction completion, the reaction mixture was diluted with CH_2_Cl_2_ (15 mL) and washed with H_2_O (10 mL). The organic layer was separated, dried over Na_2_SO_4_ and evaporated in vacuo. The desired product was purified by column chromatography.

### 3.3. General Procedure B for the Photochemical HLF Cyclization

In a glass vial, *N*-chlorosulfonamide (0.20 mmol, 1.0 equiv.), K_2_CO_3_ (33 mg, 0.24 mmol, 1.2 equiv.) and CH_2_Cl_2_ (4.0 mL) were added and the vial was sealed. The reaction mixture was degassed by bubbling with argon for 5 min. The reaction mixture was left stirring under UVA LED irradiation (370 nm) and argon atmosphere for 30 min. Afterwards, the reaction mixture was diluted with CH_2_Cl_2_ (15 mL) and washed with H_2_O (10 mL). The organic layer was separated, dried over Na_2_SO_4_ and evaporated in vacuo. Then, the residue was diluted with CH_2_Cl_2_ (4 mL) and transferred to an oven-dried pressure vessel and NaH (60% *w*/*w* in paraffin oil) (32 mg, 0.80 mmol, 4.0 equiv.) was added. The reaction mixture was heated at 100 °C and left stirring for 18 h. After reaction completion, the reaction mixture was diluted with CH_2_Cl_2_ (15 mL) and washed with H_2_O (10 mL); the organic layer was separated, dried over Na_2_SO_4_ and evaporated in vacuo. The desired product was purified by column chromatography.

2-Phenyl-1-tosylpyrrolidine (**2a**) [29]. Following General Procedure A. Yield: 76%; white solid; m.p.: 102–104 °C; ^1^H NMR (400 MHz, CDCl_3_): δ = 7.66 (2H, d, *J* = 7.6 Hz, ArH), 7.30–7.21 (7H, m, ArH), 4.78–4.76 (1H, m, NCH), 3.63–3.58 (1H, m, NC*H*H), 3.44–3.38 (1H, m, NCH*H*), 2.41 (3H, s, CH_3_), 2.00–1.93 (1H, m, CH*H*), 1.89–1.75 (2H, m, 2 × C*H*H), 1.68–1.62 (1H, m, C*H*H); ^13^C NMR (100 MHz, CDCl_3_): δ = 143.4, 143.2, 135.2, 129.7, 128.4, 127.6, 127.1, 126.2, 63.4, 49.5, 35.9, 24.1, 21.6; MS (ESI) *m*/*z* 301 [M + H]^+^.

2-Octyl-1-tosylpyrrolidine (**2b**) [30]. Following General Procedure B. Yield: 83%; yellowish oil; ^1^H NMR (400 MHz, CDCl_3_): δ = 7.71 (2H, d, *J* = 8.0 Hz, ArH), 7.29 (2H, d, *J =* 8.0 Hz, ArH), 3.61–3.55 (1H, m, NCH), 3.39–3.34 (1H, m, NC*H*H), 3.21–3.15 (1H, m, NC*H*H), 2.42 (3H, s, CH_3_), 1.85–1.71 (2H, m, CH_2_), 1.58–1.40 (4H, m, 2 × CH_2_), 1.33–1.21 (12H, m, 6 × CH_2_), 1.31 (3H, d, *J* = 6.6 Hz, CH_3_); ^13^C NMR (100 MHz, CDCl_3_): δ = 143.1, 135.0, 129.5, 127.5, 60.6, 48.8, 36.4, 31.8, 30.6, 29.5, 29.2, 26.1, 24.1, 22.6, 21.5, 14.1; MS (ESI) *m*/*z* 337 [M + H]^+^.

2-Methyl-1-tosylpyrrolidine (**2c**) [31]. Following General Procedure B. Yield: 83%; white solid; m.p.: 90–92 °C; ^1^H NMR (400 MHz, CDCl_3_): δ = 7.72 (2H, d, *J* = 8.1 Hz, ArH), 7.30 (2H, d, *J* = 8.1 Hz, ArH), 3.73–3.69 (1H, m, NCH), 3.46–3.41 (1H, m, NC*H*H), 3.18–3.12 (1H, m, NC*H*H), 2.43 (3H, s, CH_3_), 1.86–1.81 (1H, m, C*H*H), 1.74–1.65 (1H, m, C*H*H), 1.53–1.45 (1H, m, CH_2_), 1.31 (3H, d, *J* = 6.4 Hz, CH_3_); ^13^C NMR (100 MHz, CDCl_3_): δ = 143.1, 134.8, 129.5, 127.4, 56.0, 49.0, 33.4, 23.8, 22.8, 21.4; MS (ESI) *m*/*z* 239 [M + H]^+^.

2-Pentyl-1-tosylpyrrolidine (**2d**) [32]. Following General Procedure B. Yield: 56%; pale yellow oil; ^1^H NMR (400 MHz, CDCl_3_): δ = 7.71 (2H, d, *J* = 8.2 Hz, ArH), 7.30 (2H, d, *J* = 8.2 Hz, ArH), 3.64–3.56 (1H, m, NCH), 3.40–3.34 (1H, m, NC*H*H), 3.22–3.16 (1H, m, NC*H*H), 2.42 (3H, s, CH_3_), 1.84–1.71 (2H, m, CH_2_), 1.60–1.53 (2H, m, CH_2_), 1.52–1.41 (2H, m, CH_2_), 1.33–1.24 (6H, m, 3 × CH_2_), 0.89 (3H, t, *J* = 6.6 Hz, CH_3_); ^13^C NMR (100 MHz, CDCl_3_): δ = 143.1, 135.1, 129.5, 127.5, 60.6, 48.8, 36.4, 31.7, 30.6, 25.8, 24.1, 22.6, 21.5, 14.0; MS (ESI) *m*/*z* 295 [M + H]^+^.

2-Dodecyl-1-tosylpyrrolidine (**2e**) [30]. Following General Procedure B. Yield: 63%; yellow oil; ^1^H NMR (400 MHz, CDCl_3_): δ = 7.71 (2H, d, *J* = 8.1 Hz, ArH), 7.30 (2H, d, *J =* 8.1 Hz, ArH), 3.62–3.55 (1H, m, NCH), 3.40–3.34 (1H, m, NC*H*H), 3.22–3.16 (1H, m, NC*H*H), 2.42 (3H, s, CH_3_), 1.85–1.68 (2H, m, CH_2_), 1.58–1.40 (4H, m, 2 × CH_2_), 1.35–1.19 (20H, m, 10 × CH_2_), 0.88 (3H, t, *J* = 6.7 Hz, CH_3_); ^13^C NMR (100 MHz, CDCl_3_): δ = 143.1, 135.1, 129.5, 127.5, 60.6, 48.8, 36.5, 31.9, 30.6, 29.7, 29.7, 29.7, 29.6, 29.6, 29.6, 29.4, 26.2, 24.1, 22.7, 21.5, 14.1; MS (ESI) *m*/*z* 393 [M + H]^+^.

3-Methyl-1-tosylpyrrolidine (**2f**) [33]. Following General Procedure B. Yield: 48%; white solid; m.p.: 69–71 °C; ^1^H NMR (400 MHz, CDCl_3_): δ = 7.71 (2H, d, *J* = 8.0 Hz, ArH), 7.32 (2H, d, *J =* 8.0 Hz, ArH), 3.44–3.40 (1H, m, NC*H*H), 3.37–3.31 (1H, m, NC*H*H), 3.27–3.19 (1H, m, NCH*H*), 2.77–2.75 (1H, m, NC*H*H), 2.43 (3H, s, CH_3_), 2.16–2.07 (1H, m, CH), 1.94–1.86 (1H, m, C*H*H), 1.40–1.30 (1H, m, C*H*H), 0.91 (3H, d, *J* = 6.6 Hz, CH_3_); ^13^C NMR (100 MHz, CDCl_3_): δ =143.2, 134.1, 129.6, 127.5, 54.8, 47.6, 33.3, 33.3, 21.5, 17.7; MS (ESI) *m*/*z* 239 [M + H]^+^.

1-(Methylsulfonyl)-2-phenylpyrrolidine (**2g**) [19]. Following General Procedure B. Yield: 67%; white solid; m.p.: 104–106 °C; ^1^H NMR (400 MHz, CDCl_3_): δ = 7.37–7.28 (5H, m, ArH), 4.89 (1H, dd, *J* = 8.1 and 4.0 Hz, NCH), 3.70–3.65 (1H, m, NC*H*H), 3.62–3.57 (1H, m, NC*H*H), 2.68 (3H, s, CH_3_), 2.43–2.34 (1H, m, C*H*H), 2.07–1.92 (3H, m, 3 × CH*H*); ^13^C NMR (100 MHz, CDCl_3_): δ = 142.7, 128.5, 127.4, 126.3, 63.0, 49.1, 38.1, 36.2, 24.5; MS (ESI) *m*/*z* 225 [M + H]^+^.

2-Phenyl-1-(phenylsulfonyl)pyrrolidine (**2h**) [20]. Following General Procedure B. Yield: 52%; white solid; m.p.: 110–112 °C; ^1^H NMR (400 MHz, CDCl_3_): δ = 7.78 (2H, d, *J* = 8.1 Hz, ArH), 7.57 (1H, d, *J* = 7.4 Hz, ArH), 7.48 (2H, t, *J* = 7.4 Hz, ArH), 7.29–7.20 (5H, m, ArH), 4.82 (1H, dd, *J* = 7.9 and 3.7 Hz, NCH), 3.66–3.61 (1H, m, NC*H*H), 3.49–3.43 (1H, m, NC*H*H), 2.05–1.96 (1H, m, C*H*H), 1.92–1.73 (2H, m, 2 xC*H*H), 1.72–1.65 (1H, m, C*H*H); ^13^C NMR (100 MHz, CDCl_3_): δ = 142.8, 138.2, 132.5, 128.9, 128.3, 127.4, 127.0, 126.1, 63.3, 49.3, 35.8, 24.0; MS (ESI) *m*/*z* 287 [M + H]^+^.

1-(Mesitylsulfonyl)-2-phenylpyrrolidine (**2i**) [8]. Following General Procedure B. Yield: 39%; colorless oil; ^1^H NMR (400 MHz, CDCl_3_): δ = 7.09 (5H, m, ArH), 6.70 (2H, s, ArH), 4.82 (1H, dd, *J* = 7.6 and 5.6 Hz, NCH), 3.82–3.76 (1H, m, NC*H*H), 3.54–3.48 (1H, m, NC*H*H), 2.49 (6H, s, 2 × CH_3_), 2.42–2.34 (1H, m, C*H*H), 2.17 (3H, s, CH_3_), 2.07–1.94 (2H, m, 2 × C*H*H), 1.91–1.83 (1H, m, C*H*H); ^13^C NMR (100 MHz, CDCl_3_): δ = 142.2, 139.9, 133.3, 131.6, 128.3, 128.0, 126.9, 126.3, 63.3, 48.9, 37.0, 29.8, 24.7, 23.0; MS (ESI) *m*/*z* 329 [M + H]^+^.

## 4. Conclusions

In conclusion, we have developed a catalyst-free, UVA-promoted Hofmann–Löffler–Freytag cyclization for the synthesis of pyrrolidines from the more stable class of halogenated amines, namely *N*-chlorosulfonamides. This protocol provides a mild and sustainable approach for achieving remote C(sp^3^)–H functionalization without the need for transition-metal catalysts or photocatalysts. Moreover, this work introduces a practical strategy for the facile generation of nitrogen-centered radicals, which could be further exploited in a broad range of HLF-type transformations reported in the literature.

## Figures and Tables

**Table 1:** Irradiation source optimization in the photochemical HLF reaction of **1a**.

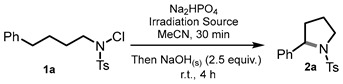		
Entry	Irradiation Source (nm)	Yield 2a (%)
1	370	56
2	370 2nd generation	19
3	390	15
4	400	41
5	427	0
6	440	0
7	456	0
8	467	0
9	525	0
10	640	0

The reaction performed with *N*-chloro-4-methyl-*N*-(4-phenylbutyl)benzenesulfonamide (**1a**, 67 mg, 0.20 mmol) and Na_2_HPO_4_ (34 mg, 0.24 mmol, 1.2 equiv.) under irradiation (Kessil lamps) in MeCN (4 mL) for 30 min. Then, NaOH (20 mg, 0.50 mmol, 2.5 equiv.) was added and the reaction was left stirring for 4 h at room temperature. Yield of **2a** was determined after purification by column chromatography.

**Table 2:** Solvent optimization for the photochemical HLF reaction of **1a**.

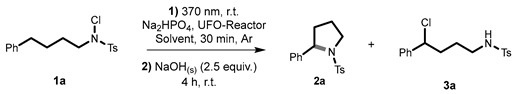			
Entry	Solvent	Yield (%) ^a^	
		2a	3a
1	MeCN	74 (56)	
2	CH_2_Cl_2_	37 (35)	45
3	EtOAc	22 (21)	56
4	MeOH	66 (51)	16
5	H_2_O	-	-
6	Toluene	30 (23)	38
7	DCE	32 (33)	41
8	CHCl_3_	53 (43)	20
9	2-Me-THF	32	31

The reaction performed with *N*-chloro-4-methyl-*N*-(4-phenylbutyl)benzenesulfonamide (**1a**, 67 mg, 0.20 mmol) and Na_2_HPO_4_ (34 mg, 0.24 mmol, 1.2 equiv.) under irradiation at 370 nm (Kessil PR160L) in solvent (4 mL) for 30 min. Then, NaOH (20 mg, 0.50 mmol, 2.5 equiv.) was added and the reaction was left stirring for 4 h at room temperature. ^a^ Conversion was determined by ^1^H-NMR. Yield of isolated product by column chromatography is shown in parenthesis.

**Table 3:** Base optimization for the photochemical HLF reaction of **1a**.

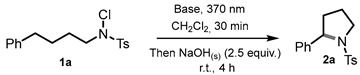		
Entry	Base	Yield 2a (%) ^a^
1	Na_2_HPO_4_	37 (35)
2	Cs_2_CO_3_	40
3	K_2_CO_3_	86 (76)
4 ^b^	K_2_CO_3_	70 (68)
5 ^c^	K_2_CO_3_	33
6	Na_2_CO_3_	80 (70)
7	Pyridine	38
8	Et_3_N	traces
9	-	59 (53)

The reaction performed with *N*-chloro-4-methyl-*N*-(4-phenylbutyl)benzenesulfonamide (**1a**, 67 mg, 0.20 mmol) and base (0.24 mmol, 1.2 equiv.) under irradiation at 370 nm (Kessil PR160L) in CH_2_Cl_2_ (4 mL) for 30 min. Then, NaOH (20 mg, 0.50 mmol, 2.5 equiv.) was added and the reaction was left stirring for 4 h at room temperature. ^a^ Conversion was determined by ^1^H-NMR. Yield of isolated product by column chromatography is shown in parenthesis. ^b^ Reaction was performed with MeCN instead of CH_2_Cl_2_. ^c^ Reaction was performed with 3.5 equv. of NaOH.

**Table 4:** Optimization for the cyclization of unreactive substrates.

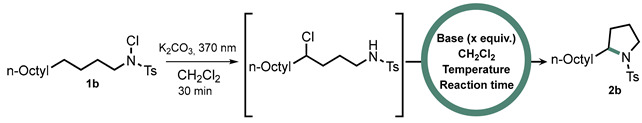				
Entry	Base (Equiv.)	Reaction Time (h)	Temperature (°C)	Conversion (%) ^a^
1	NaOH (2.5)	4	25	traces
2	DBU (2.5)	5	40	48
3	DBU (2.5)	18	40	53
4	DBU (2.5)	5	70	56
5	DBU (5.0)	24	70	80
6	DBU (7.5)	40	70	83
7	Et_3_N (2.5)	18	70	8
8	NaOH (2.5)	18	70	36
9	NaH (2.5)	18	70	58
10	NaH (4.0)	18	70	62
11	NaH (4.0)	18	100	95 (83)

The reaction performed with *N*-chloro-*N*-dodecyl-4-methylbenzenesylfonamide (**1b**, 75 mg, 0.20 mmol) and K_2_CO_3_ (33 mg, 0.24 mmol, 1.2 equiv.) under irradiation at 370 nm (Kessil PR160L 370 nm) in CH_2_Cl_2_ (4 mL) for 30 min. Then, the base (2.5–7.5 equiv.) was added and the reaction was left stirring for 5–40 h. ^a^ Conversion was determined by ^1^H-NMR. Yield of isolated product by column chromatography is shown in parenthesis.

## Data Availability

All data supporting this study are included in the article and Supplementary Materials.

## Supplementary Materials

The following supporting information can be downloaded at: https://www.mdpi.com/article/10.3390/molecules31111963/s1. Ref. [34] is cited in the Supplementary Materials.
