# Supplementary material for: Photochemical Catalyst-Free Synthesis of Pyrrolidines via a Hofmann–Loffler–Freytag Reaction
Source: Molecules. 2026 Jun 5;31(11):1963. doi: 10.3390/molecules31111963 (PMC13257522; doi:10.3390/molecules31111963)
Supplement: Supplementary file 1 [file molecules-31-01963-s001.zip › molecules-4347942-supplementary.pdf]

Photochemical Catalyst-Free Synthesis of Pyrrolidines via a Hofmann-Loffler-Freytag Reaction

Athina S. J. Shkembi[‡], Luca Pasqualon[‡], Stamatis K. Serviou, Manos V. G. Lantzanakis and Christoforos G. Kokotos*

Laboratory of Organic Chemistry, Department of Chemistry,

National and Kapodistrian University of Athens,

Panepistimiopolis, Athens 15771, Greece

SUPPORTING INFORMATION

	Page
**Control Experiments**	**S2**
**Synthesis of Starting Materials**	**S3**
**Reaction setup for the HLF Reaction**	**S8**
**UV-Vis Studies**	**S9**
**References**	**S10**
**NMR Spectra**	**S11**

Control experiments

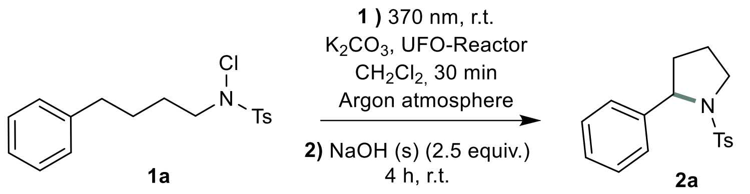

Entry	Deviation from standard conditions	Conversion (%)[a]
1	No irradiation	0
2	No irradiation and heating of reaction mixture at 60 ˚C	0

The reaction was performed with *N*-chloro-4-methyl-*N*-(4-phenylbutyl)benzenesulfonamide (**1a**) (67 mg, 0.20 mmol, 1.0 equiv.), K_2CO_3 (33 mg, 0.24 mmol, 1.2 equiv.) in CH_2Cl_2 (4 mL) without irradiation for 30 min, under an argon atmosphere. Afterwards, solid NaOH (20 mg, 0.50 mmol, 2.5 equiv.) was added to the reaction mixture and it was left stirring for 4 hours. [a] Conversion was determined by ^{1}H-NMR.

Synthesis of Starting Materials

General Procedure for the Synthesis of *N*-Chloro Sulfonamides

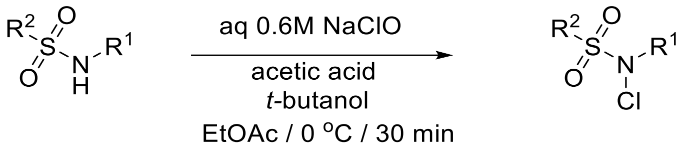

In a round-bottom flask with a magnetic stirrer, *t*-butanol (0.22 g, 0.3 mL, 3.00 mmol, 10.0 equiv.), acetic acid (0.36 g, 0.4 mL, 6.00 mmol, 20.0 equiv.) and sulfonamide (0.30 mmol, 1.0 equiv.) were dissolved in ethyl acetate (10 mL). The reaction mixture was cooled at 0 °C. An aqueous solution of NaClO (0.6 M, 7.6 mL, 4.50 mmol, 15.0 equiv.) was added dropwise and the reaction mixture was left stirring for 30 min. The reaction mixture was diluted with EtOAc (20 mL) and washed with a saturated aqueous solution of $NaHCO_3$ (10 mL) and brine (10 mL), dried over Na_2SO_4, filtered, and evaporated to dryness. The product was used without further purification.

N-Chloro-4-methyl-*N*-(4-phenylbutyl)benzenesulfonamide (1a)[1]

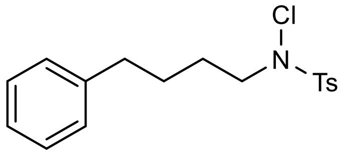

White solid; m.p.: 64-66 °C; **Yield:** 91%; **^{1}H NMR** (400 MHz, $CDCl_3$): δ = 7.80 (2H, d, *J* = 7.7 Hz, ArH), 7.38 (2H, d, *J* = 7.7 Hz, ArH), 7.30-7.26 (2H, m, ArH), 7.20-7.16 (3H, m, ArH), 3.25-3.21 (2H, m, NCH_2), 2.66-2.63 (2H, m, CH_2), 2.46 (3H, s, CH_3), 1.72-1.70 (4H, m, 2 x CH_2); **^{13}C NMR** (100 MHz, $CDCl_3$): δ = 145.4, 141.8, 129.8, 129.7, 129.6, 128.4, 128.4, 125.9, 56.4, 35.2, 27.7, 26.5, 21.7; **MS (ESI)** m/z 337 $[M+H]^+$.

N-Chloro-N-dodecyl-4-methylbenzenesulfonamide (1b)

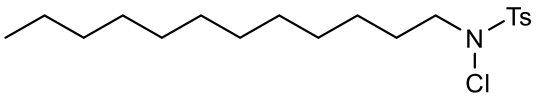

Colorless oil; **Yield:** 95%; **^{1}H NMR** (400 MHz, $CDCl_3$): δ = 7.81 (2H, d, J = 7.9 Hz, ArH), 7.37 (2H, d, J = 7.9 Hz, ArH), 3.21 (2H, t, J = 6.8 Hz, NCH_2), 2.46 (3H, s, CH_3), 1.69-1.62 (2H, m, CH_2), 1.37-1.20 (18H, m, 9 x CH_2), 0.87 (3H, t, J = 6.5 Hz, CH_3); **^{13}C NMR** (100 MHz, $CDCl_3$): δ = 145.3, 130.0, 129.7, 129.6, 56.7, 31.9, 29.6, 29.6, 29.5, 29.5, 29.3, 29.0, 27.0, 26.1, 22.7, 21.7, 14.1; **MS (ESI)** m/z 373 $[M+H]^+$.

N-Chloro-4-methyl-N-pentylbenzenesulfonamide (1c)[1]

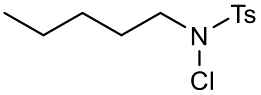

Colorless oil; **Yield:** 93%; **^{1}H NMR** (400 MHz, $CDCl_3$): δ = 7.80 (2H, d, J = 7.8 Hz, ArH), 7.37 (2H, d, J = 7.8 Hz, ArH), 3.20 (2H, m, NCH_2), 2.46 (3H, s, CH_3), 1.69-1.62 (2H, m, CH_2), 1.35-1.29 (4H, m, 2 x CH_2), 0.89 (3H, t, J = 6.1 Hz, CH_3); **^{13}C NMR** (100 MHz, $CDCl_3$): δ = 145.3, 129.6, 129.5, 127.0, 56.6, 28.1, 26.6, 22.1, 21.6, 13.8; **MS (ESI)** m/z 275 $[M+H]^+$.

N-Chloro-4-methyl-N-nonylbenzenesulfonamide (1d)

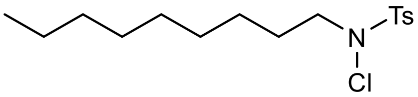

Colorless oil; **Yield:** 60%; **^{1}H NMR** (400 MHz, $CDCl_3$): δ = 7.81 (2H, d, J = 7.5 Hz, ArH), 7.38 (2H, d, J = 7.5 Hz, ArH), 3.20 (2H, t, J = 6.3 Hz, NCH_2), 2.46 (3H, s, CH_3), 1.69-1.62 (2H, m, CH_2), 1.35-1.21 (12H, m, 6 x CH_2), 0.86 (3H, t, J = 6.5 Hz, CH_3); **^{13}C NMR** (100 MHz, $CDCl_3$):

δ = 145.2, 129.9, 129.7, 129.6, 56.7, 31.8, 29.4, 29.2, 29.1, 27.0, 26.0, 22.7, 21.7, 14.1; **MS (ESI)** m/z 331 $[M+H]^+$.

N-Chloro-N-hexadecyl-4-methylbenzenesulfonamide (1e)

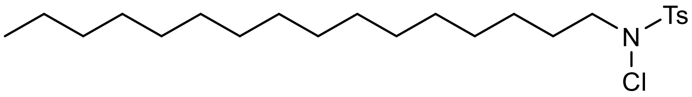

Colorless oil; **Yield:** 96%; **^{1}H NMR** (400 MHz, $CDCl_3$): δ = 7.81 (2H, d, *J* = 8.2 Hz, ArH), 7.37 (2H, d, *J* = 8.2 Hz, ArH), 3.21 (2H, t, *J* = 6.9 Hz, NCH_2), 2.46 (3H, s, CH_3), 1.69-1.62 (2H, m, CH_2), 1.37-1.20 (26H, m, 13 x CH_2), 0.87 (3H, t, *J* = 6.7 Hz, CH_3); **^{13}C NMR** (100 MHz, $CDCl_3$): δ = 145.3, 130.0, 129.7, 129.6, 56.7, 31.9, 29.7, 29.7, 29.7, 29.7, 29.6, 29.6, 29.5, 29.5, 29.4, 29.1, 27.1, 26.1, 22.7, 21.7, 14.1; **MS (ESI)** m/z 429 $[M+H]^+$.

***N*-Chloro-*N*-isopentyl-4-methylbenzenesulfonamide (1f)**

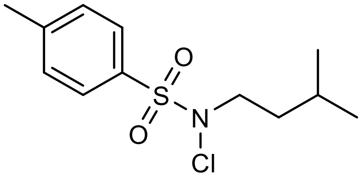

Pale yellow oil; **Yield:** 88%; **^{1}H NMR** (400 MHz, $CDCl_3$): δ = 7.83 (2H, d, *J* = 8.1 Hz, ArH), 7.40 (2H, d, *J* = 8.1 Hz, ArH), 3.26 (2H, t, *J* = 6.9 Hz, NCH_2), 2.48 (3H, s, CH_3), 1.77-1.67 (1H, m, CH), 1.59-1.54 (2H, m, CH_2), 0.93 (6H, d, *J* = 6.7 Hz, 2 x CH_3); **^{13}C NMR** (100 MHz, $CDCl_3$): δ = 145.4, 129.8, 129.7, 129.6, 55.1, 35.7, 25.1, 22.3, 21.7; **MS (ESI)** m/z 275 $[M+H]^+$.

N-Chloro-N-(4-phenylbutyl)methanesulfonamide (1g)[1]

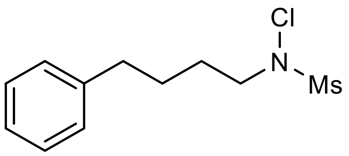

Pale yellow oil; **Yield:** 89%; ^{1}**H NMR** (400 MHz, $CDCl_3$): δ = 7.34-7.31 (2H, m, ArH), 7.25-7.21 (3H, m, ArH), 3.48 (2H, t, *J* = 5.9 Hz, NCH_2), 3.08 (3H, s, CH_3), 2.66-2.63 (2H, t, *J* = 6.9 Hz, CH_2), 1.84-1.74 (4H, m, 2 x CH_2); ^{13}**C NMR** (100 MHz, $CDCl_3$): δ = 141.6, 128.3, 128.3, 125.8, 55.6, 35.1, 33.6, 27.5, 26.5; **MS (ESI)** m/z 261 $[M+H]^{+}$.

N-Chloro-N-(4-phenylbutyl)benzenesulfonamide (1h)

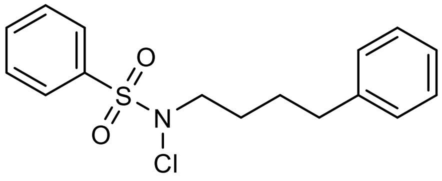

Pale yellow oil; **Yield:** 88%; ^{1}**H NMR** (400 MHz, $CDCl_3$): δ = 7.96 (2H, d, *J* = 7.6 Hz, ArH), 7.43 (1H, t, *J* = 7.6 Hz, ArH), 7.62 (2H, t, *J* = 7.6 Hz, ArH), 7.43-7.29 (3H, m, ArH), 7.23-7.19 (2H, m, ArH), 3.28 (2H, t, *J* = 5.7 Hz, NCH_2), 2.68 (2H, t, *J* = 6.7 Hz, CH_2), 1.80-1.70 (4H, m, 2 x CH_2); ^{13}**C NMR** (100 MHz, $CDCl_3$): δ = 141.8, 134.3, 132.9, 129.5, 129.1, 128.4, 128.4, 125.9, 56.5, 35.2, 27.7, 26.5; **MS (ESI)** m/z 325 $[M+H]^{+}$.

N-Chloro-N-(4-phenyllbutyl)mesitylsulfonamide (1i)

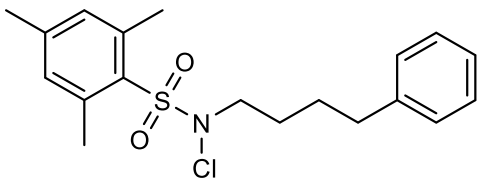

Yellow oil; **Yield:** 88%; **^{1}H NMR** (400 MHz, $CDCl_3$): δ = 7.32-7.26 (2H, m, ArH), 7.22-7.16 (3H, m, ArH), 7.00 (2H, s, ArH), 3.55 (2H, t, *J* = 6.6 Hz, NCH_2), 2.69 (6H, s, 2 x CH_3), 2.64 (2H, t, *J* = 7.5 Hz, $ArCH_2$), 2.33 (3H, s, CH_3), 1.86-1.79 (2H, m, CH_2), 1.74-1.67 (2H, m, CH_2); **^{13}C NMR** (100 MHz, $CDCl_3$): δ = 144.2, 141.9, 141.8, 132.0, 129.0, 128.4, 128.4, 125.9, 52.6, 35.2, 27.9, 26.4, 23.0, 21.2; **MS (ESI)** m/z 365 $[M+H]^+$.

Reaction setup for the HLF Reaction

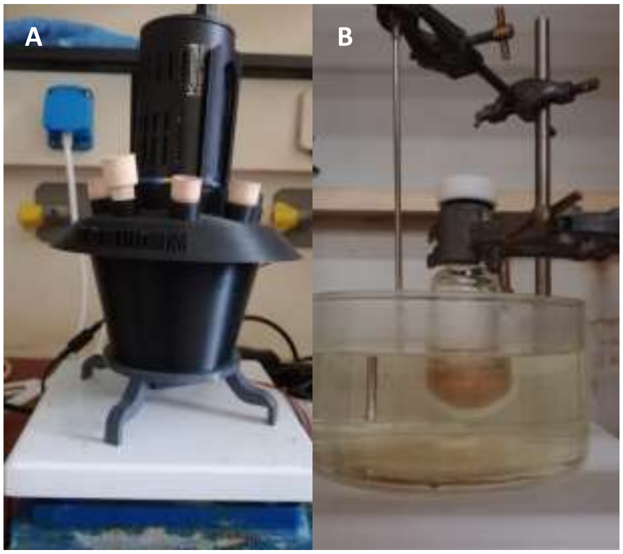

Figure S1. A) Reaction setup for the photochemical step, **B)** Reaction setup for the cyclization process.

UV-Vis Studies

UV-Vis studies were conducted with 4 mL of CH_2Cl_2 and 0.20 mmol (67 mg) of *N*-chloro-4-methyl-*N*-(4-phenylbutyl)benzenesulfonamide (**1a**).

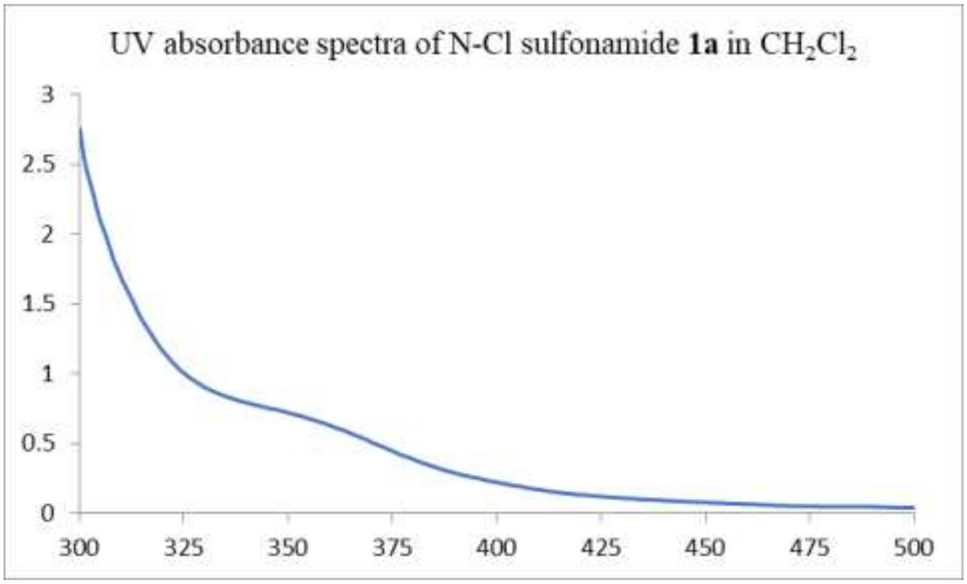

Figure S2. UV-Vis absorbance spectra of **1a** in CH_2Cl_2 (50 mM).

References

1. D. Chang, R. Zhao, C. Wei, Y. Yao, Y. Liu, L. Shi, *J. Org. Chem.* **2018**, *83*, 3305-3315.

NMR Spectra

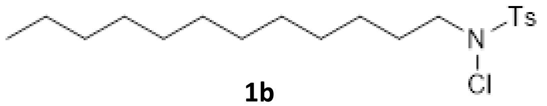

1b

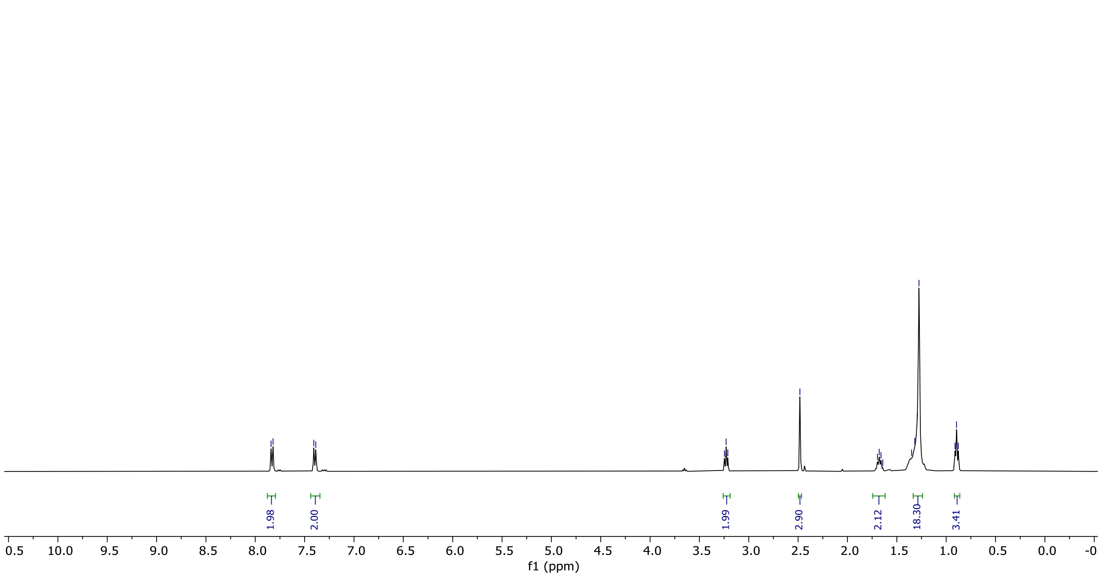

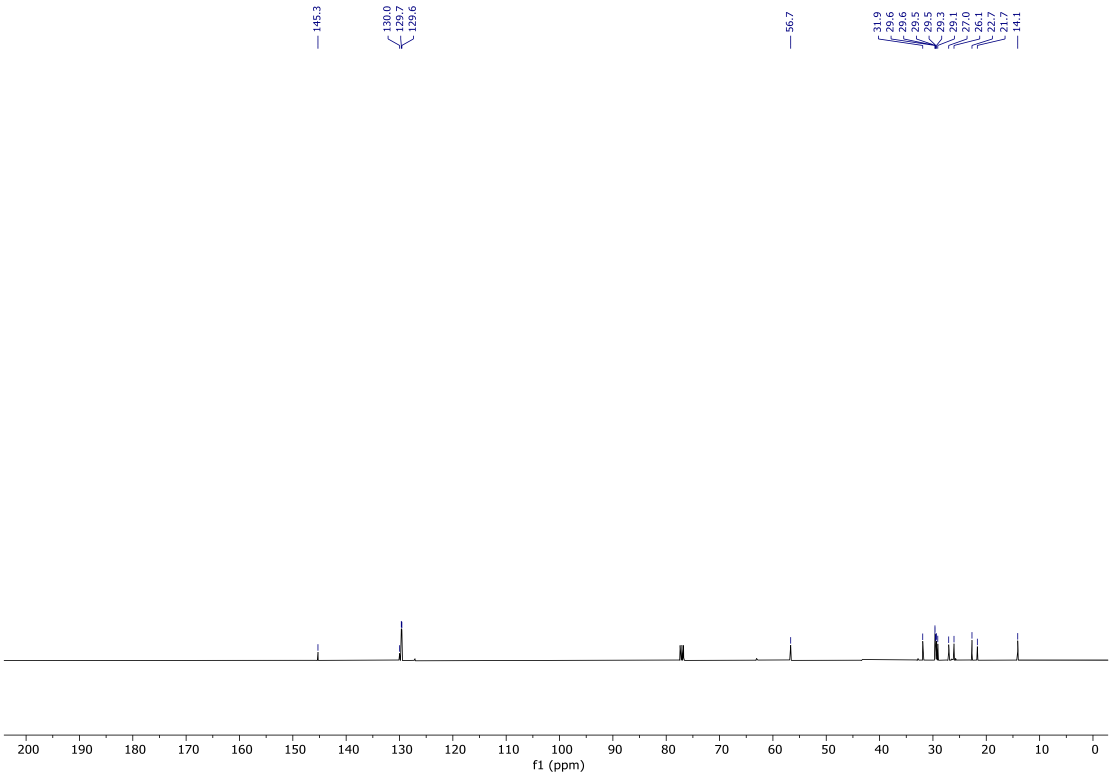

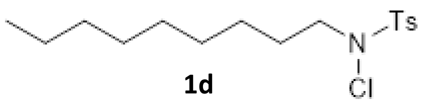
Ts
N
Cl

1d

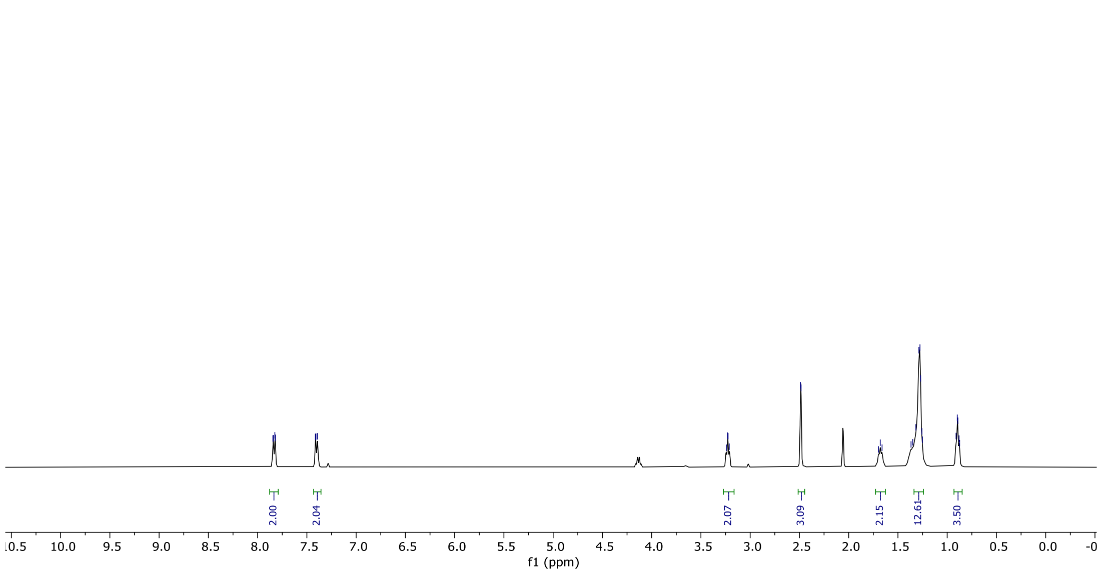
7.84 7.84 7.83 7.82 7.41 7.41 7.39
3.24 3.23 3.22 3.21
2.49 2.48
1.70 1.68 1.66 1.37 1.35 1.32 1.29 1.28 1.27 1.26 1.25 0.91 0.90 0.89 0.88 0.87
2.00 2.04 2.07 3.09 2.15 12.61 3.50
10.5 10.0 9.5 9.0 8.5 8.0 7.5 7.0 6.5 6.0 5.5 5.0 4.5 4.0 3.5 3.0 2.5 2.0 1.5 1.0 0.5 0.0
f1 (ppm)

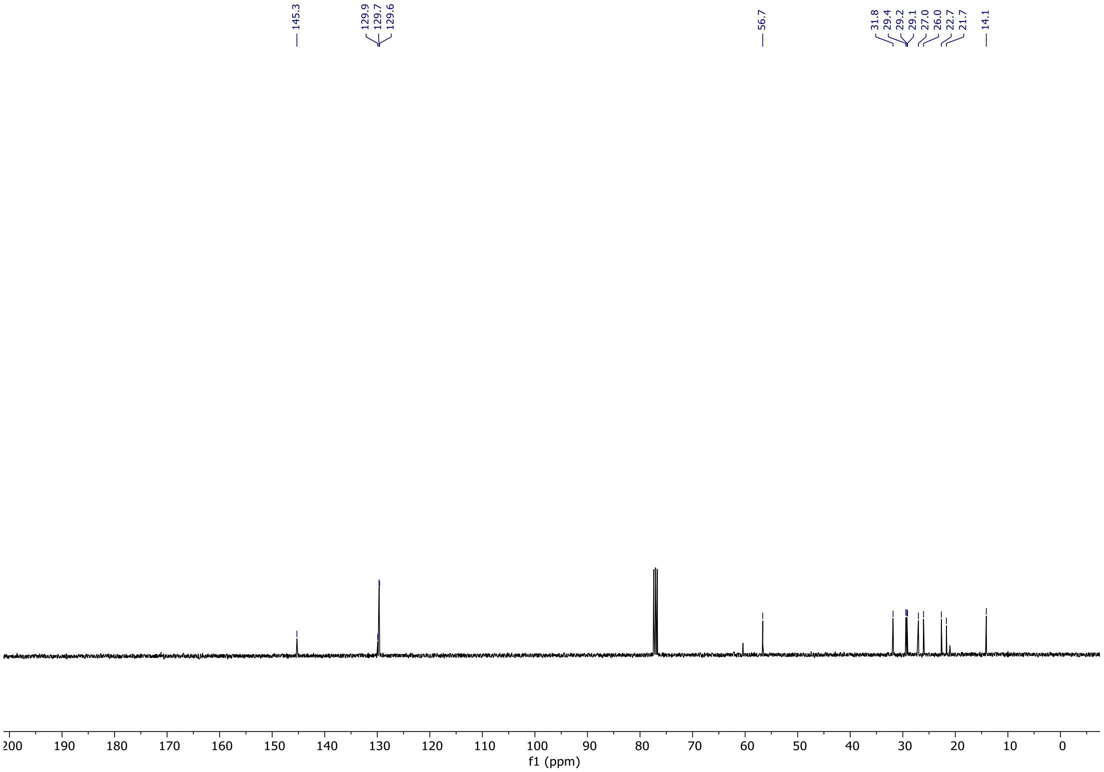
145.3
129.9 129.7 129.6
56.7
31.8 29.4 29.2 29.1 27.0 26.0 22.7 21.7
14.1
200 190 180 170 160 150 140 130 120 110 100 90 80 70 60 50 40 30 20 10 0
f1 (ppm)

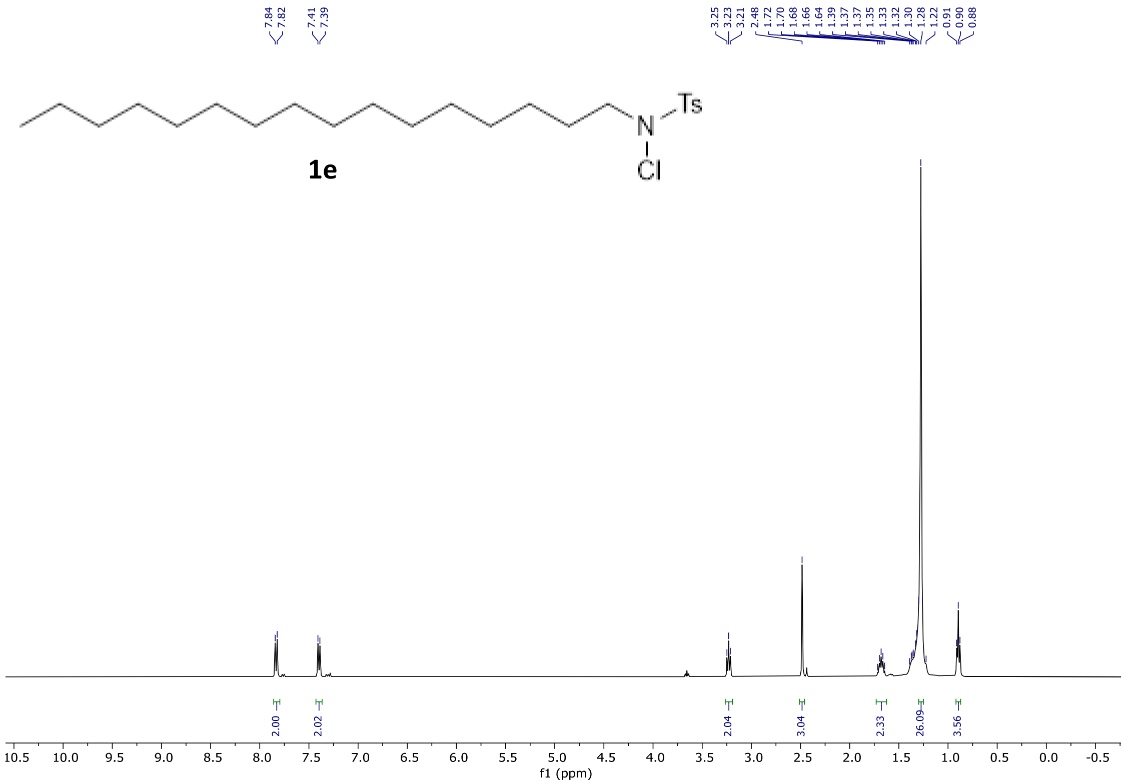

7.84
7.82
7.41
7.39
3.25
3.23
3.21
2.48
1.72
1.70
1.68
1.66
1.64
1.39
1.37
1.37
1.35
1.33
1.32
1.30
1.28
1.22
0.91
0.90
0.88
Ts
N
Cl
1e
2.00
2.02
2.04
3.04
2.33
26.09
3.56
10.5 10.0 9.5 9.0 8.5 8.0 7.5 7.0 6.5 6.0 5.5 5.0 4.5 4.0 3.5 3.0 2.5 2.0 1.5 1.0 0.5 0.0 -0.5
f1 (ppm)

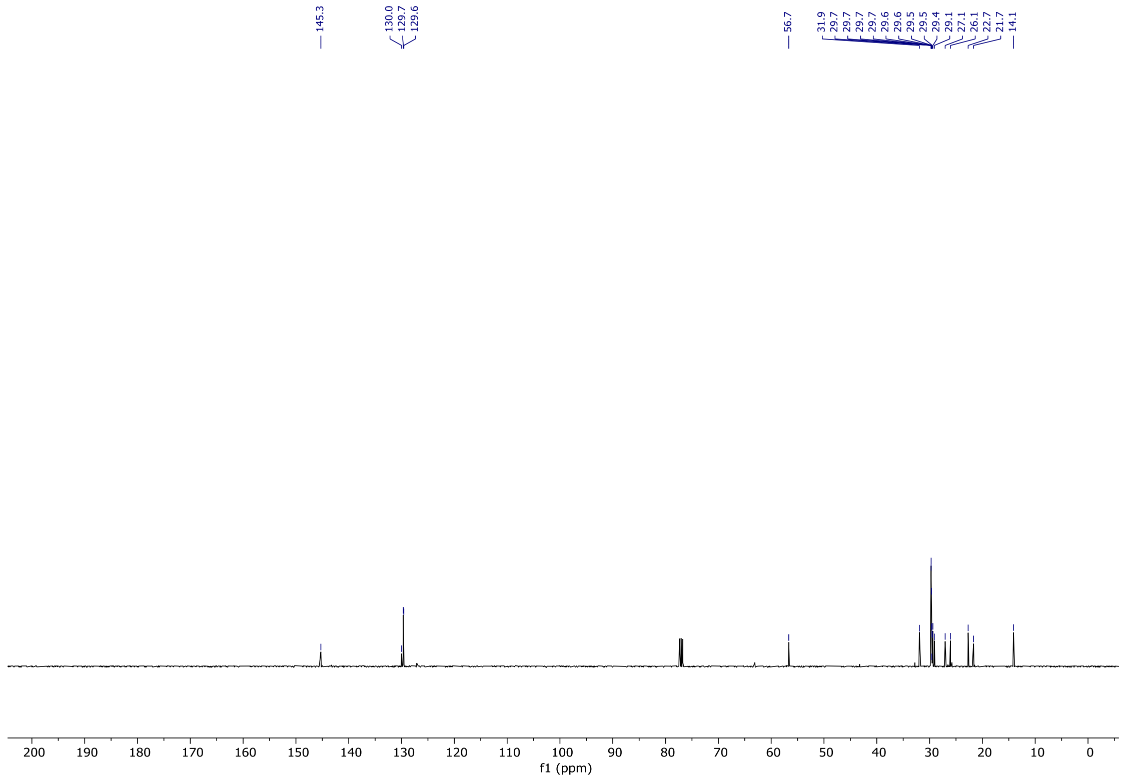

145.3
130.0
129.7
129.6
56.7
31.9
29.7
29.7
29.7
29.7
29.6
29.6
29.5
29.5
29.4
29.1
27.1
26.1
22.7
21.7
14.1
200 190 180 170 160 150 140 130 120 110 100 90 80 70 60 50 40 30 20 10 0
f1 (ppm)

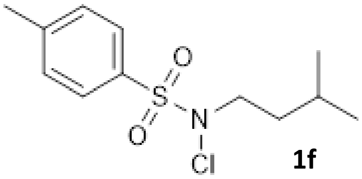
O
S
N
O
Cl

1f

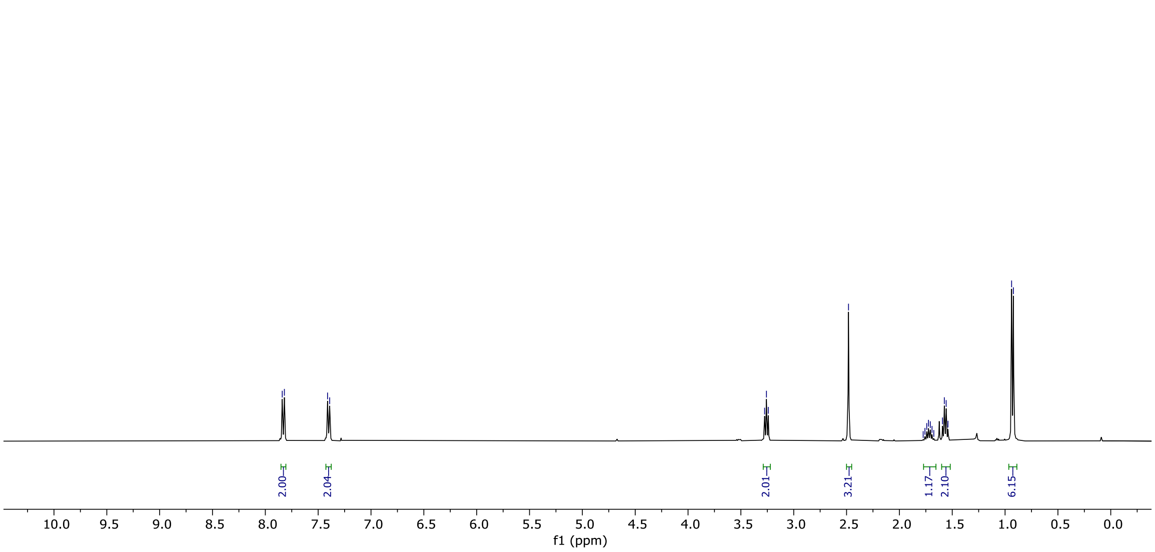
7.84
7.82
7.41
7.39
3.28
3.26
3.24
2.48
1.78
1.76
1.74
1.72
1.71
1.69
1.67
1.59
1.57
1.56
1.54
0.94
0.92
2.00
2.04
2.01
3.21
1.17
2.10
6.15
f1 (ppm)

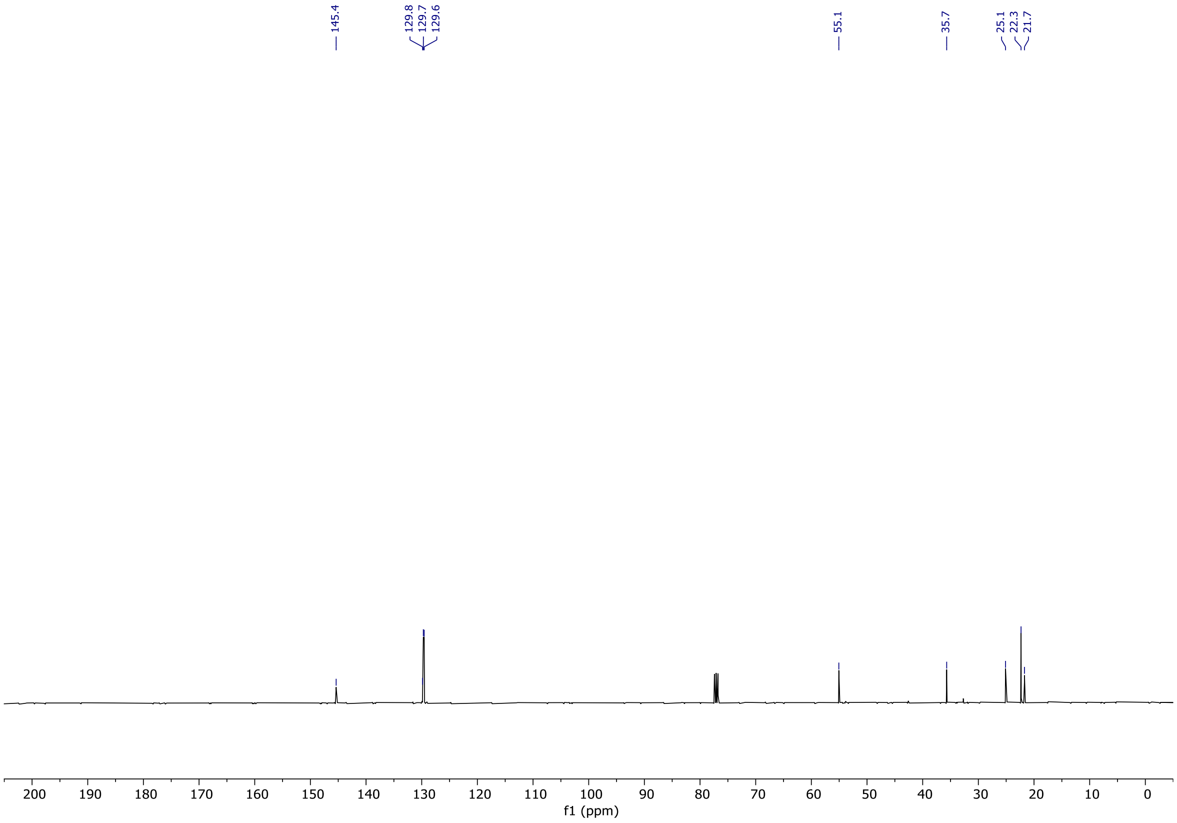
145.4
129.8
129.7
129.6
55.1
35.7
25.1
22.3
21.7
f1 (ppm)

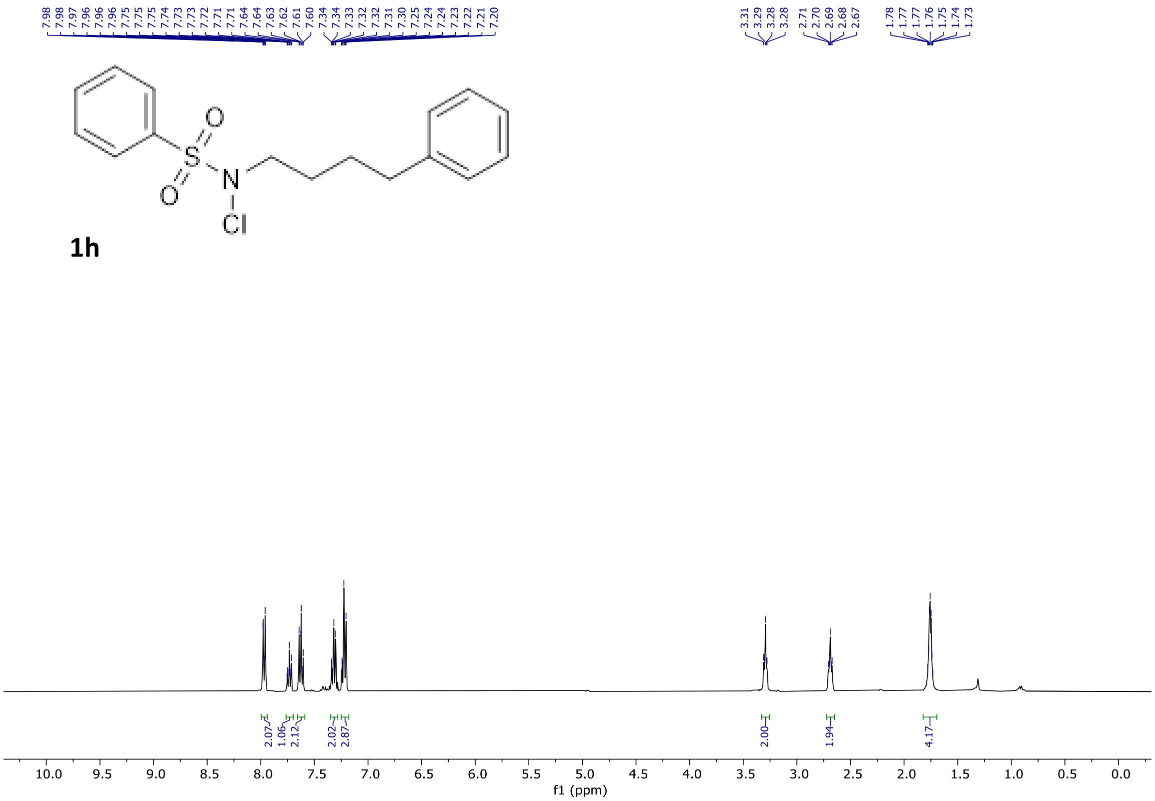
7.98 7.98 7.97 7.96 7.96 7.96 7.75 7.75 7.75 7.74 7.73 7.73 7.72 7.71 7.71 7.64 7.64 7.63 7.62 7.61 7.60 7.34 7.34 7.33 7.32 7.32 7.31 7.30 7.25 7.24 7.24 7.23 7.22 7.21 7.20
3.31 3.29 3.28 3.28 2.71 2.70 2.69 2.68 2.67 1.78 1.77 1.77 1.76 1.75 1.74 1.73
O
S
N
O
Cl
1h
2.07 1.06 2.12 2.02 2.87 2.00 1.94 4.17
10.0 9.5 9.0 8.5 8.0 7.5 7.0 6.5 6.0 5.5 5.0 4.5 4.0 3.5 3.0 2.5 2.0 1.5 1.0 0.5 0.0
f1 (ppm)

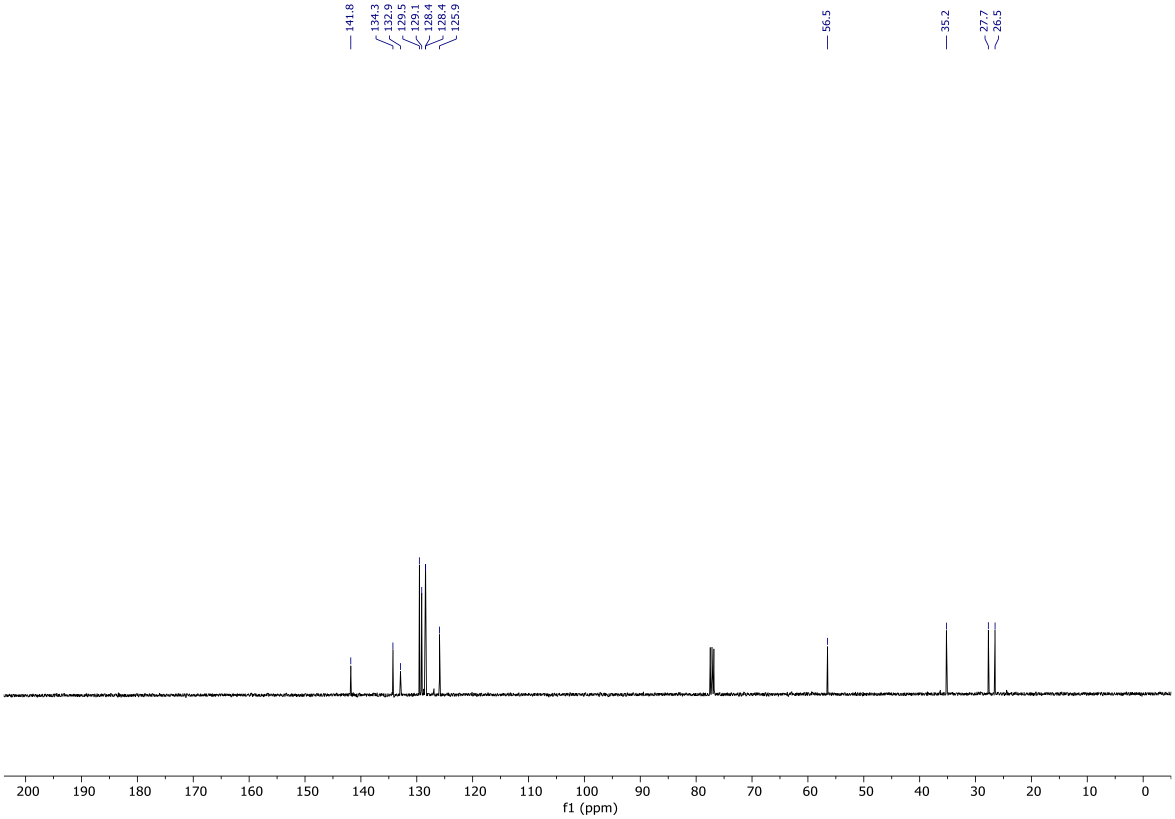
141.8 134.3 132.9 129.5 129.1 128.4 128.4 125.9 56.5 35.2 27.7 26.5
200 190 180 170 160 150 140 130 120 110 100 90 80 70 60 50 40 30 20 10 0
f1 (ppm)

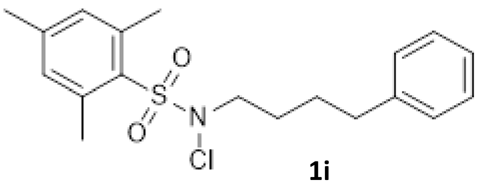
O
S
N
O
Cl

1i

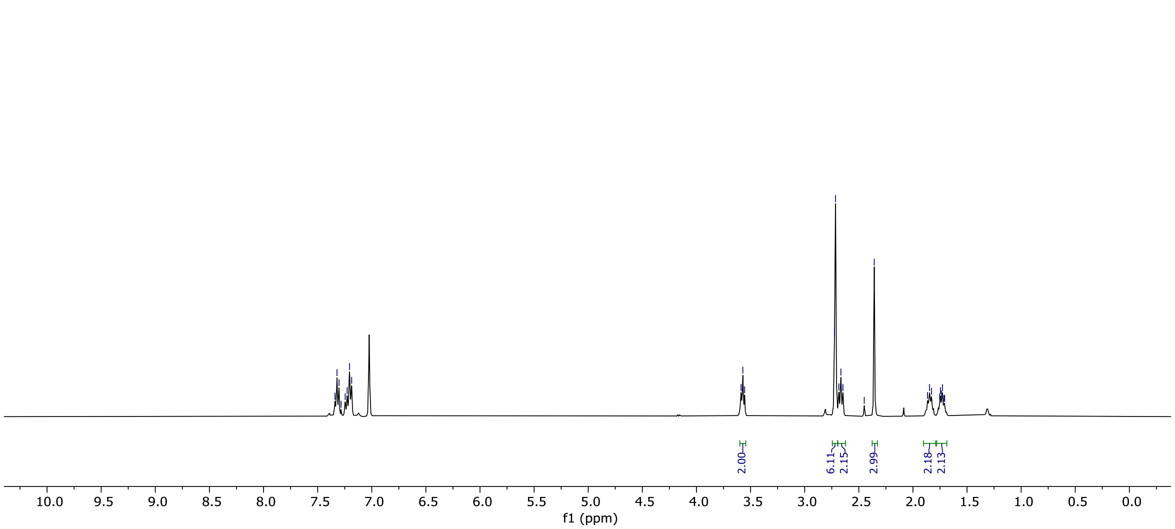
7.34
7.32
7.30
7.28
7.25
7.23
7.21
7.19
3.59
3.57
3.55
2.72
2.72
2.68
2.67
2.65
2.45
2.36
1.86
1.85
1.83
1.75
1.75
1.73
1.73
1.71
1.71
2.00
6.11
2.15
2.99
2.18
2.13
10.0 9.5 9.0 8.5 8.0 7.5 7.0 6.5 6.0 5.5 5.0 4.5 4.0 3.5 3.0 2.5 2.0 1.5 1.0 0.5 0.0
f1 (ppm)

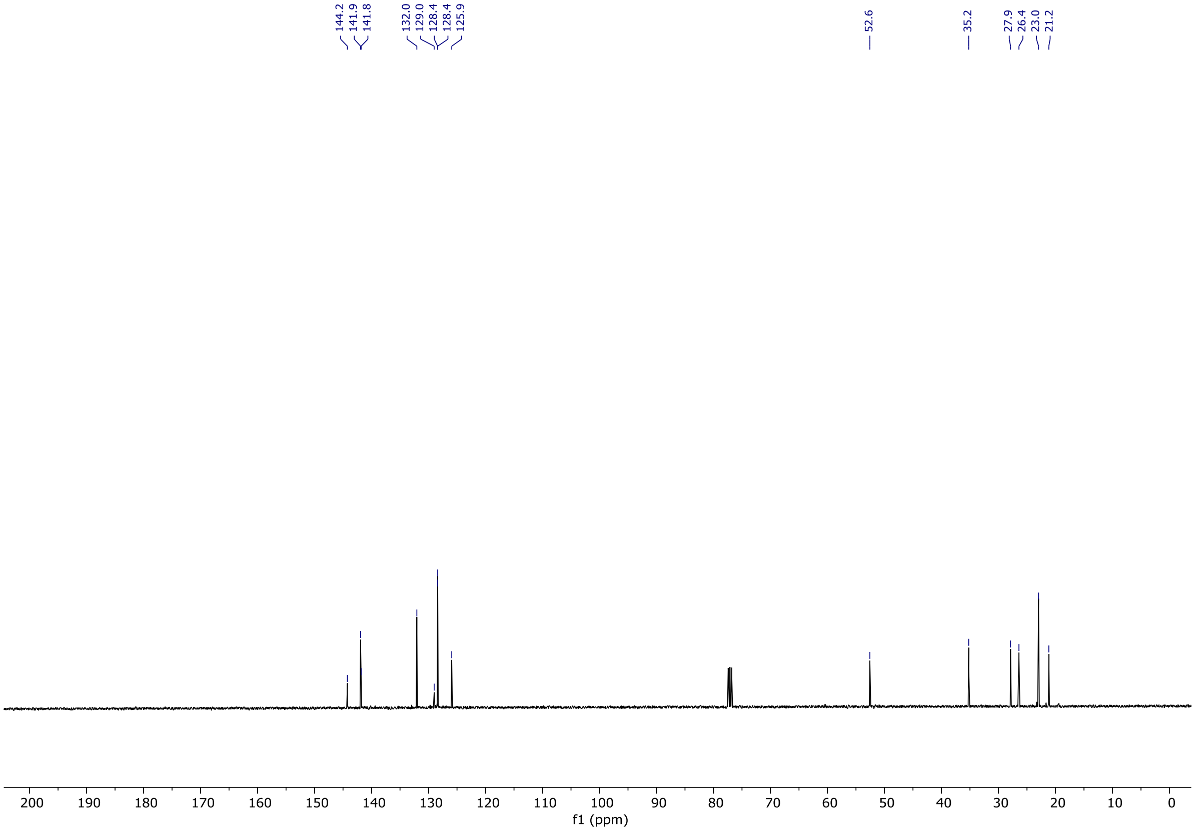
144.2
141.9
141.8
132.0
129.0
128.4
128.4
125.9
52.6
35.2
27.9
26.4
23.0
21.2
200 190 180 170 160 150 140 130 120 110 100 90 80 70 60 50 40 30 20 10 0
f1 (ppm)

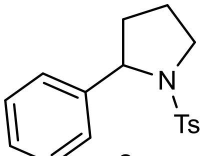
N
Ts

2a

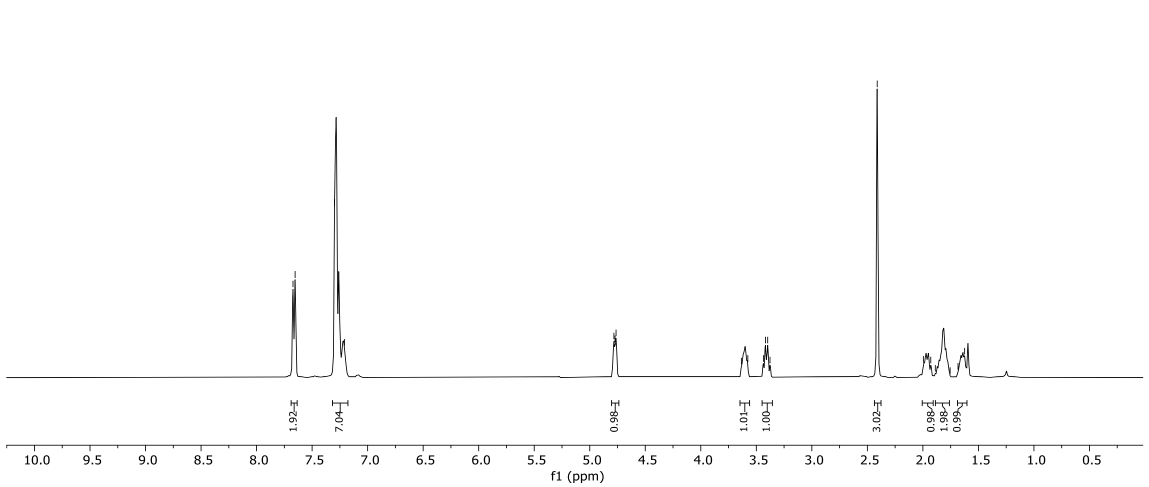
7.67
7.65
7.30
7.21
4.78
4.77
4.76
3.63
3.58
3.44
3.42
3.40
3.38
2.41
2.00
1.93
1.89
1.75
1.68
1.62
1.92
7.04
0.98
1.01
1.00
3.02
0.98
1.98
0.99
10.0 9.5 9.0 8.5 8.0 7.5 7.0 6.5 6.0 5.5 5.0 4.5 4.0 3.5 3.0 2.5 2.0 1.5 1.0 0.5
f1 (ppm)

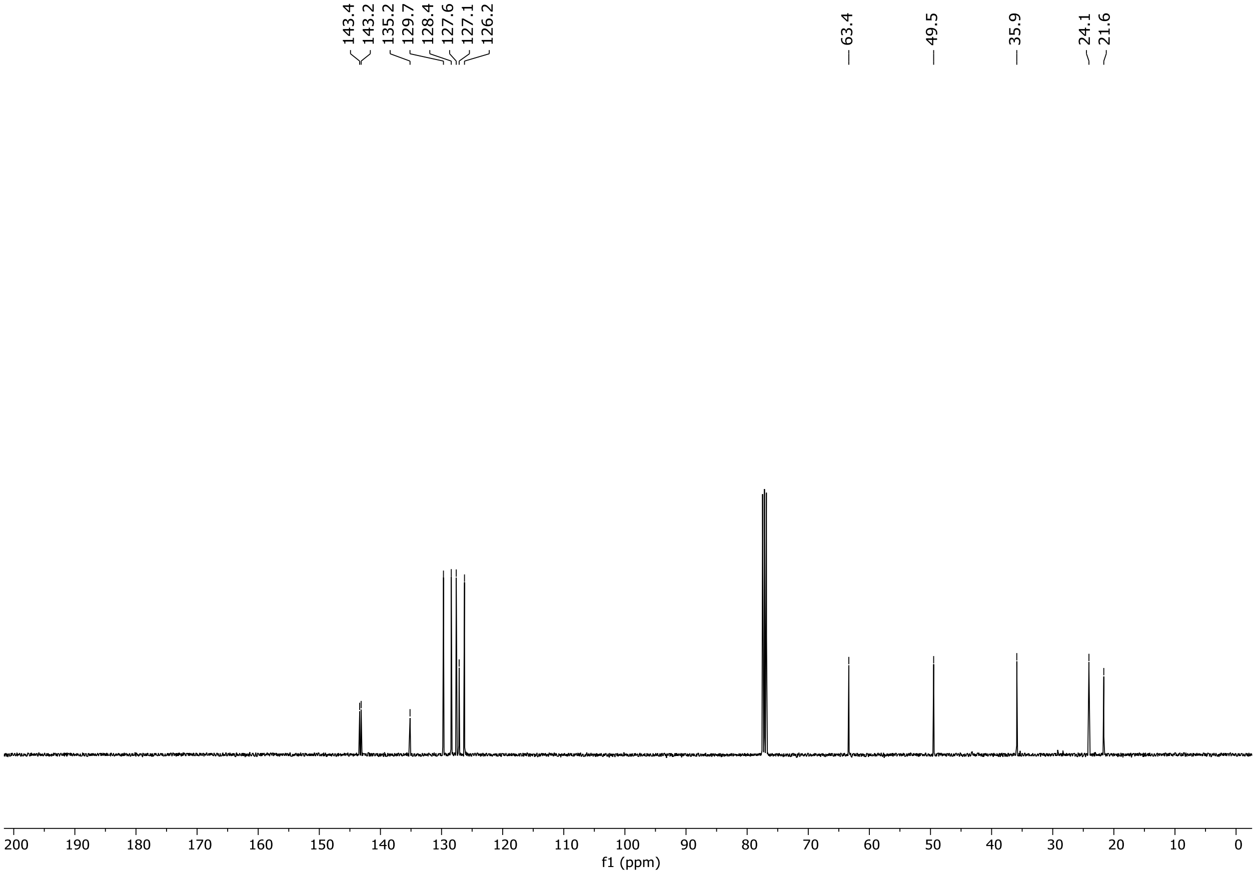
143.4
143.2
135.2
129.7
128.4
127.6
127.1
126.2
63.4
49.5
35.9
24.1
21.6
200 190 180 170 160 150 140 130 120 110 100 90 80 70 60 50 40 30 20 10 0
f1 (ppm)

2b

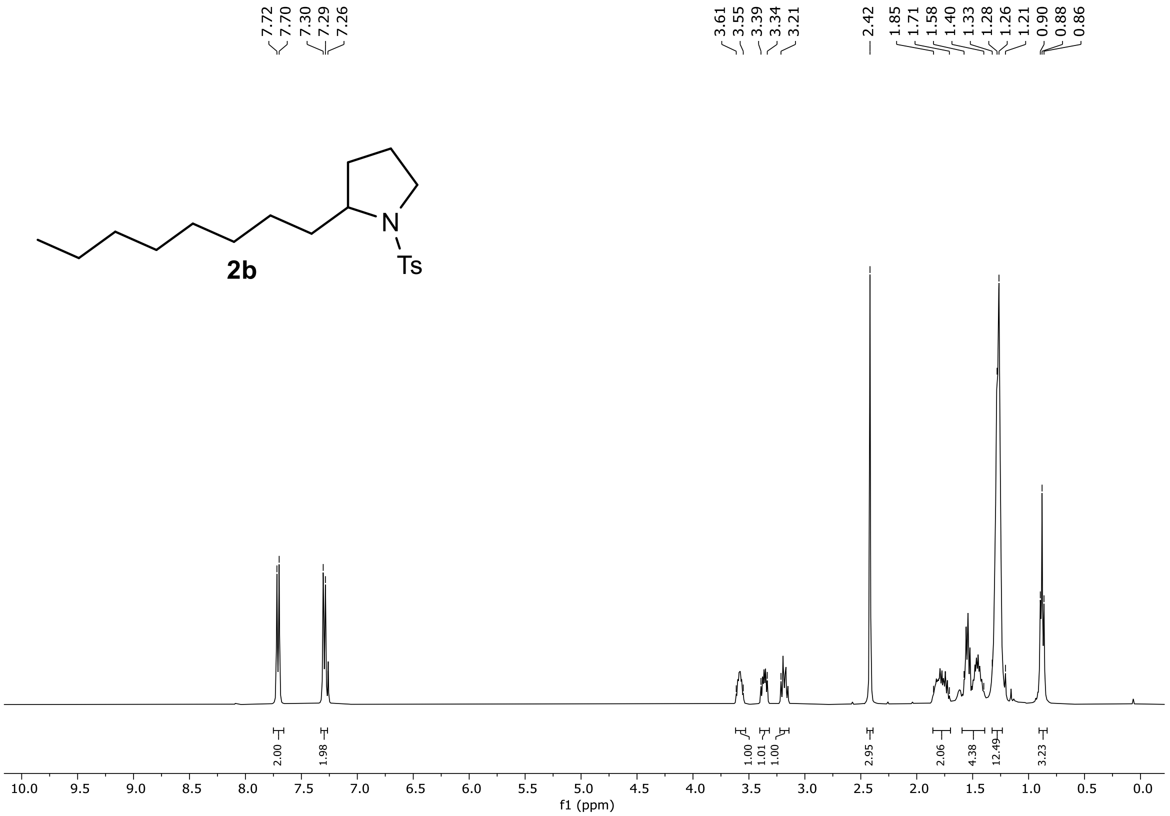
7.72
7.70
7.30
7.29
7.26
3.61
3.55
3.39
3.34
3.21
2.42
1.85
1.71
1.58
1.40
1.33
1.28
1.26
1.21
0.90
0.88
0.86
2.00
1.98
1.00
1.01
1.00
2.95
2.06
4.38
12.49
3.23
f1 (ppm)

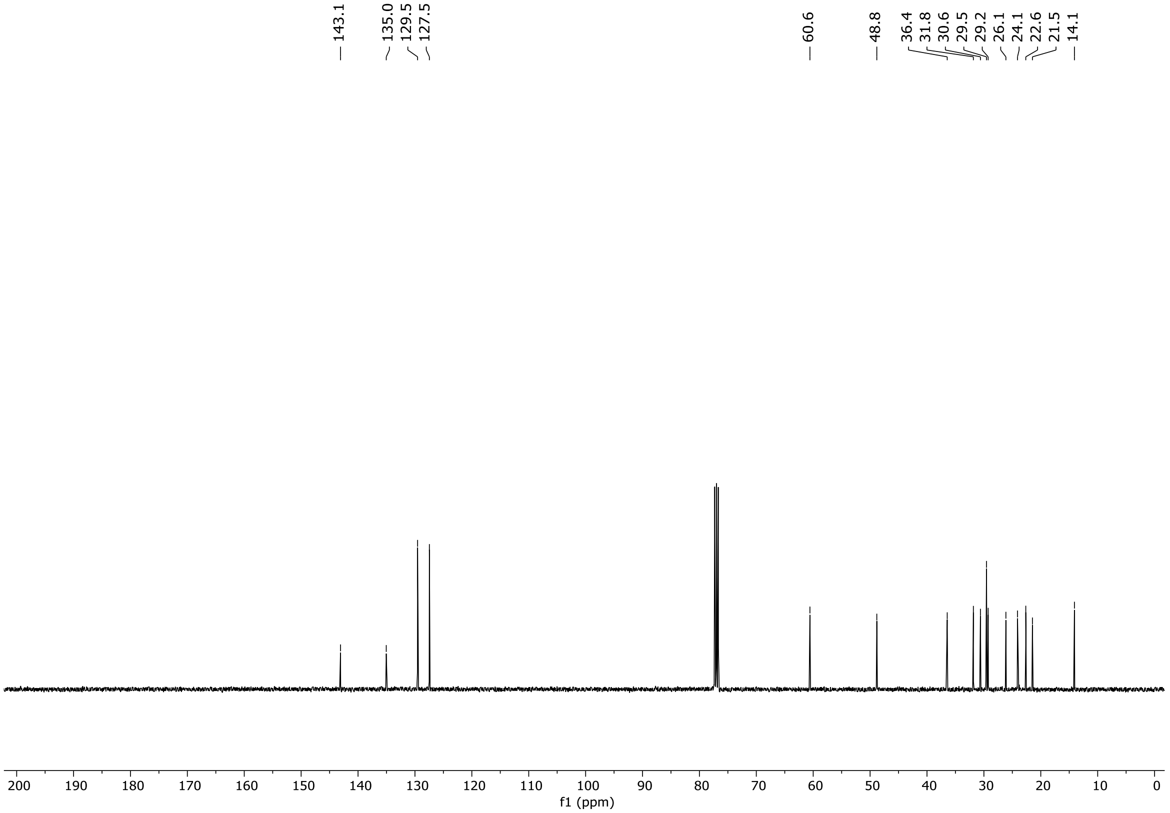
143.1
135.0
129.5
127.5
60.6
48.8
36.4
31.8
30.6
29.5
29.2
26.1
24.1
22.6
21.5
14.1
f1 (ppm)

2c

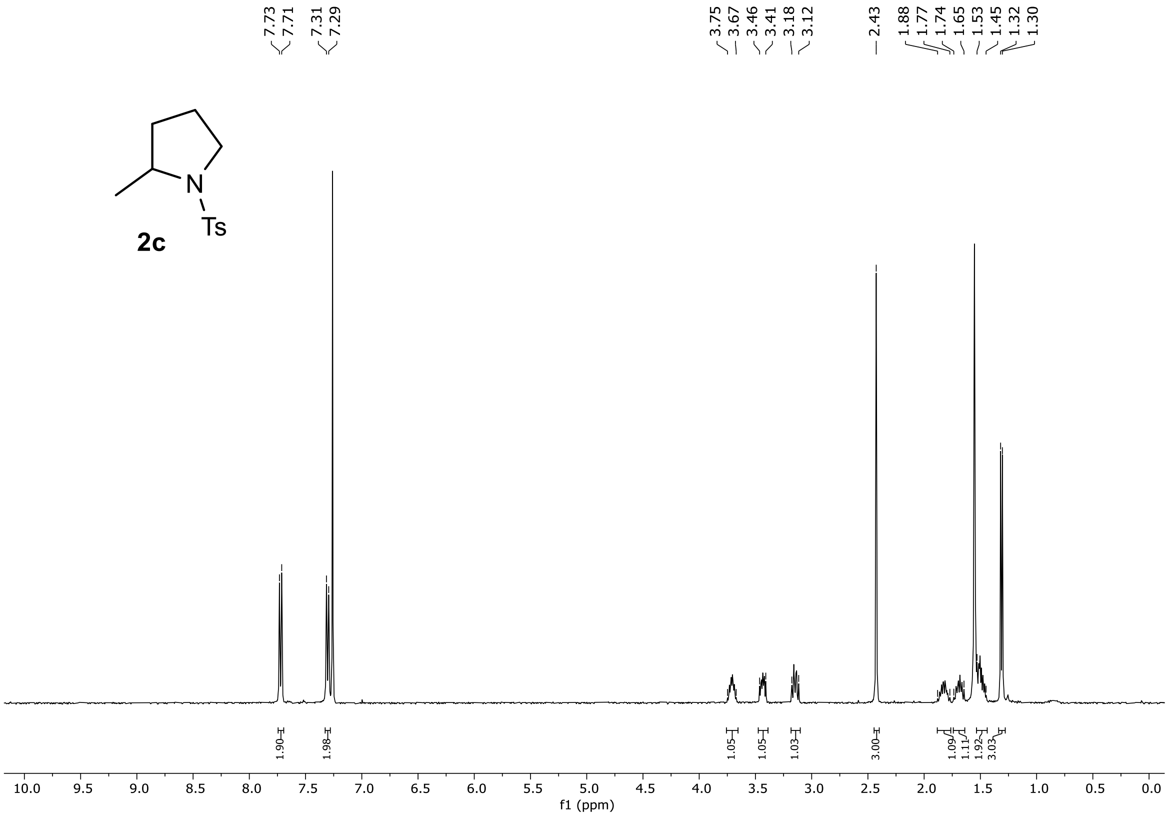
N
Ts
7.73
7.71
7.31
7.29
3.75
3.67
3.46
3.41
3.18
3.12
2.43
1.88
1.77
1.74
1.65
1.53
1.45
1.32
1.30
1.90
1.98
1.05
1.05
1.03
3.00
1.09
1.11
1.92
3.03
f1 (ppm)

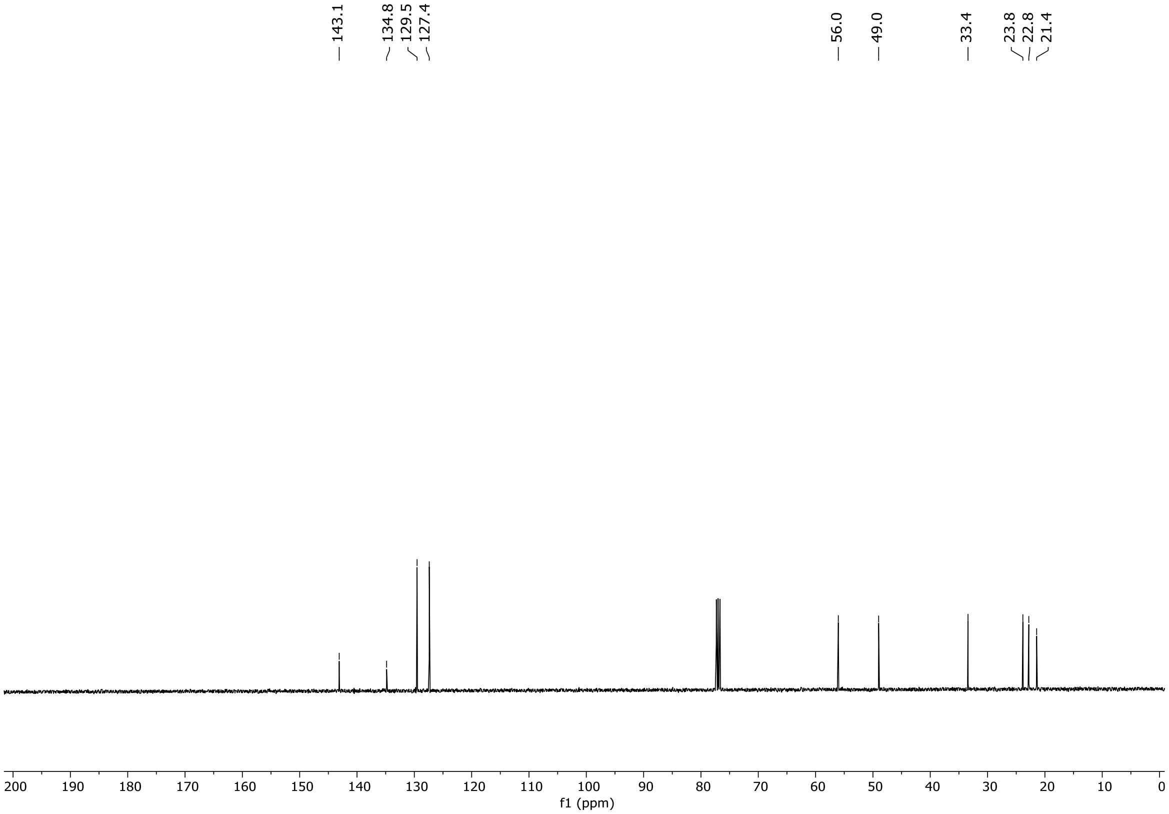
143.1
134.8
129.5
127.4
56.0
49.0
33.4
23.8
22.8
21.4
f1 (ppm)

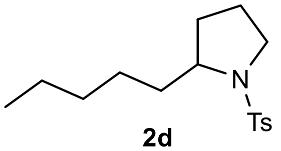
N
Ts

2d

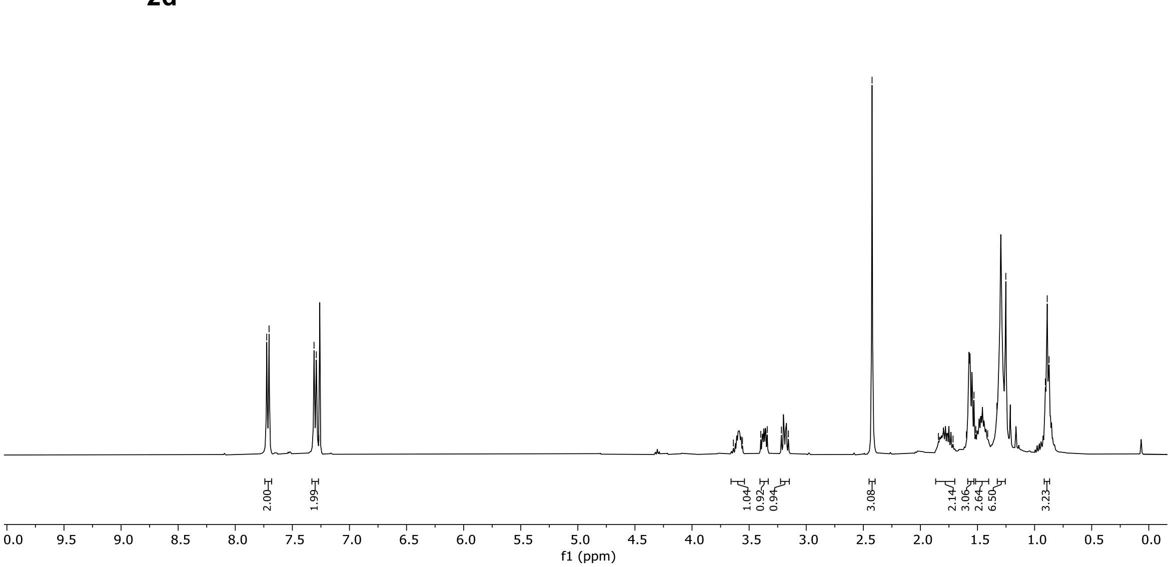
7.72
7.70
7.31
7.29
7.27
3.64
3.56
3.40
3.34
3.22
3.16
2.42
1.84
1.71
1.60
1.53
1.52
1.41
1.33
1.25
0.91
0.89
0.87
2.00
1.99
1.04
0.92
0.94
3.08
2.14
3.06
2.64
6.50
3.23
0.0 9.5 9.0 8.5 8.0 7.5 7.0 6.5 6.0 5.5 5.0 4.5 4.0 3.5 3.0 2.5 2.0 1.5 1.0 0.5 0.0
f1 (ppm)

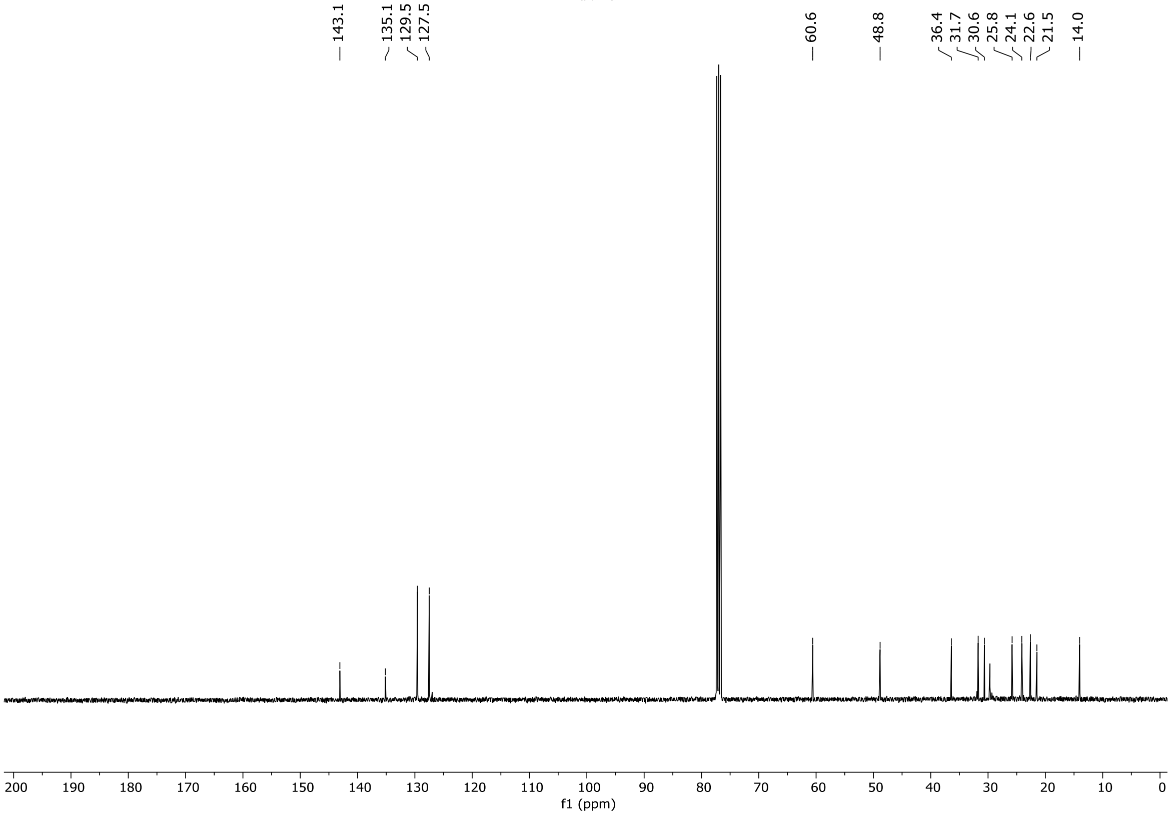
143.1
135.1
129.5
127.5
60.6
48.8
36.4
31.7
30.6
25.8
24.1
22.6
21.5
14.0
200 190 180 170 160 150 140 130 120 110 100 90 80 70 60 50 40 30 20 10 0
f1 (ppm)

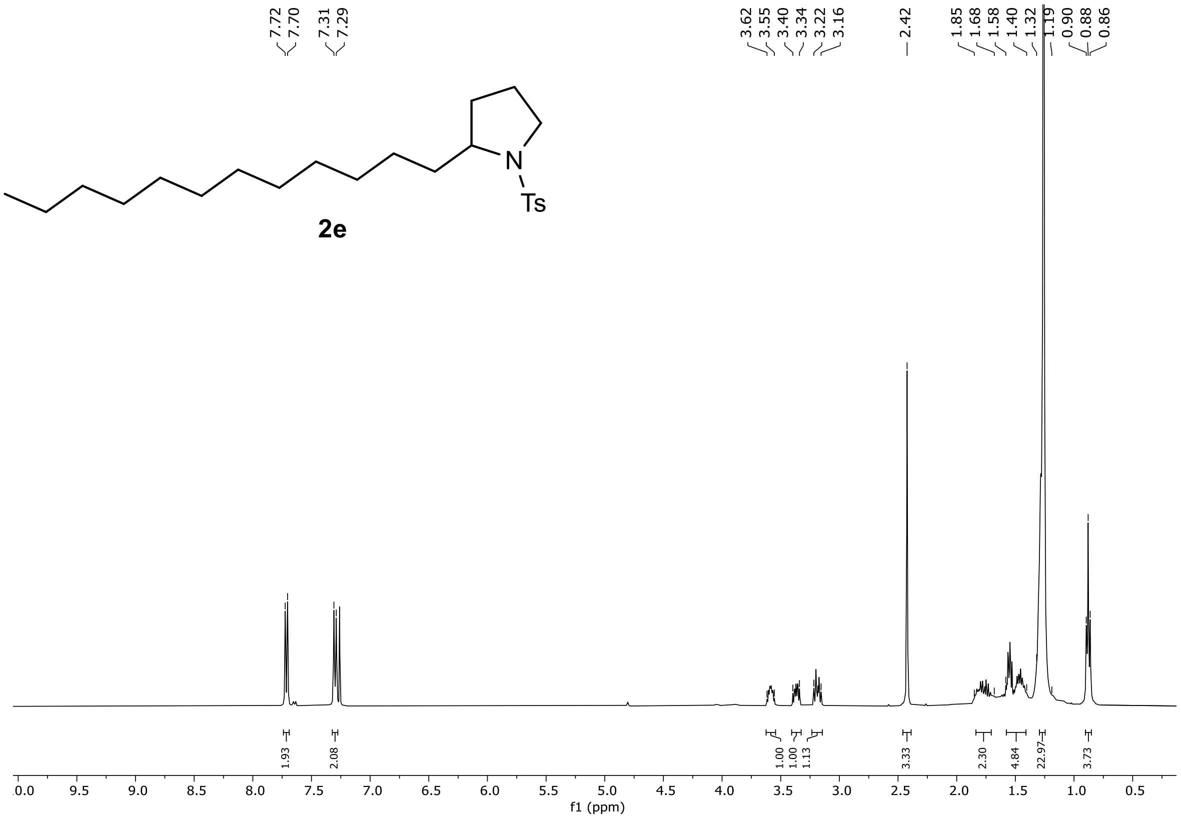

2e

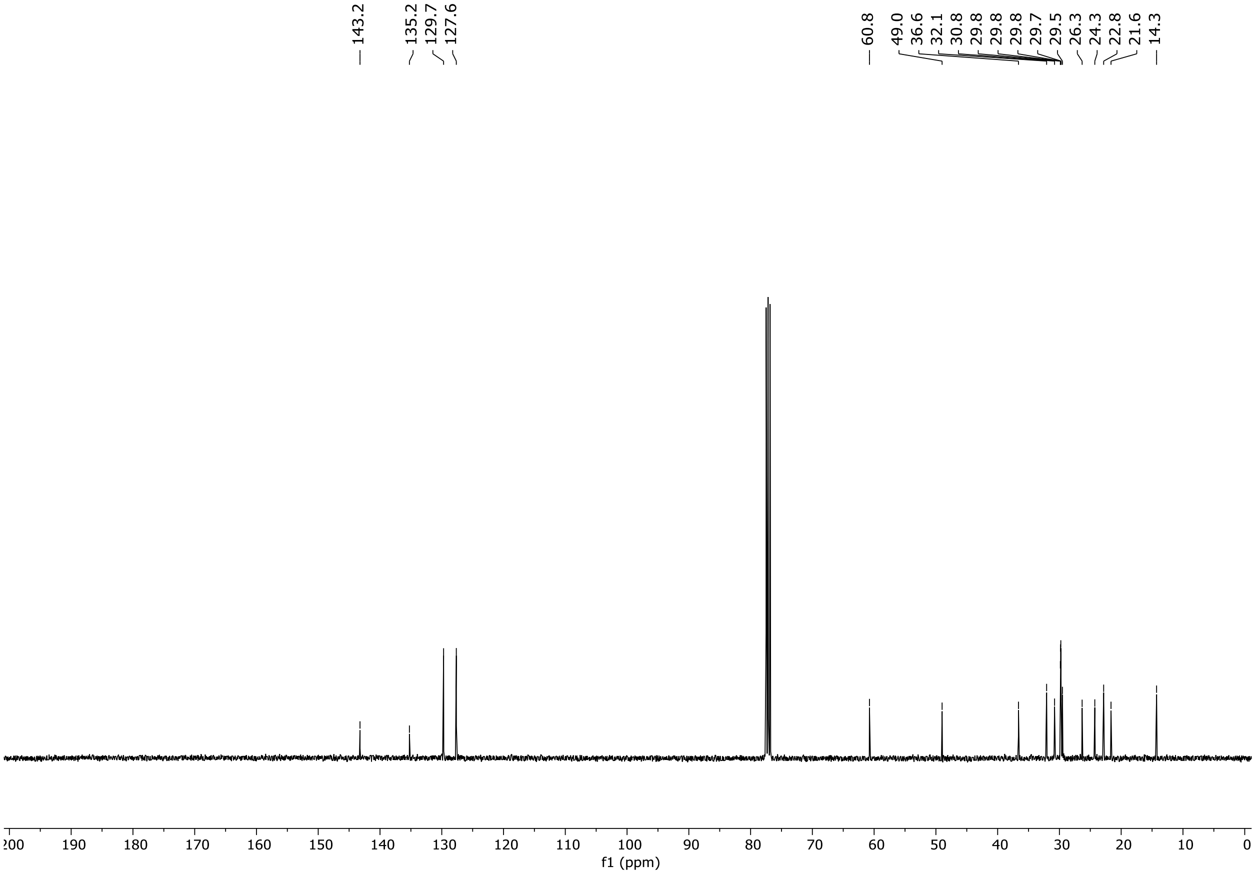

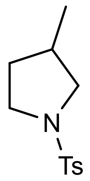
N
Ts

2f

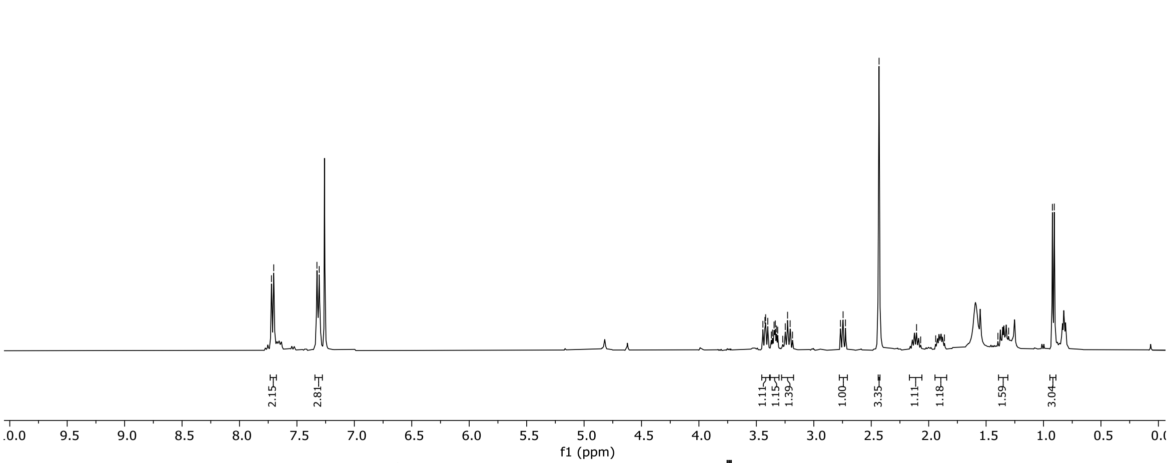
7.72
7.70
7.33
7.31
3.44
3.43
3.42
3.40
3.37
3.36
3.35
3.34
3.32
3.31
3.27
3.25
3.23
3.21
3.19
2.77
2.75
2.72
2.43
2.11
2.07
1.94
1.86
1.40
1.30
0.92
0.91
2.15
2.81
1.11
1.15
1.39
1.00
3.35
1.11
1.18
1.59
3.04
f1 (ppm)

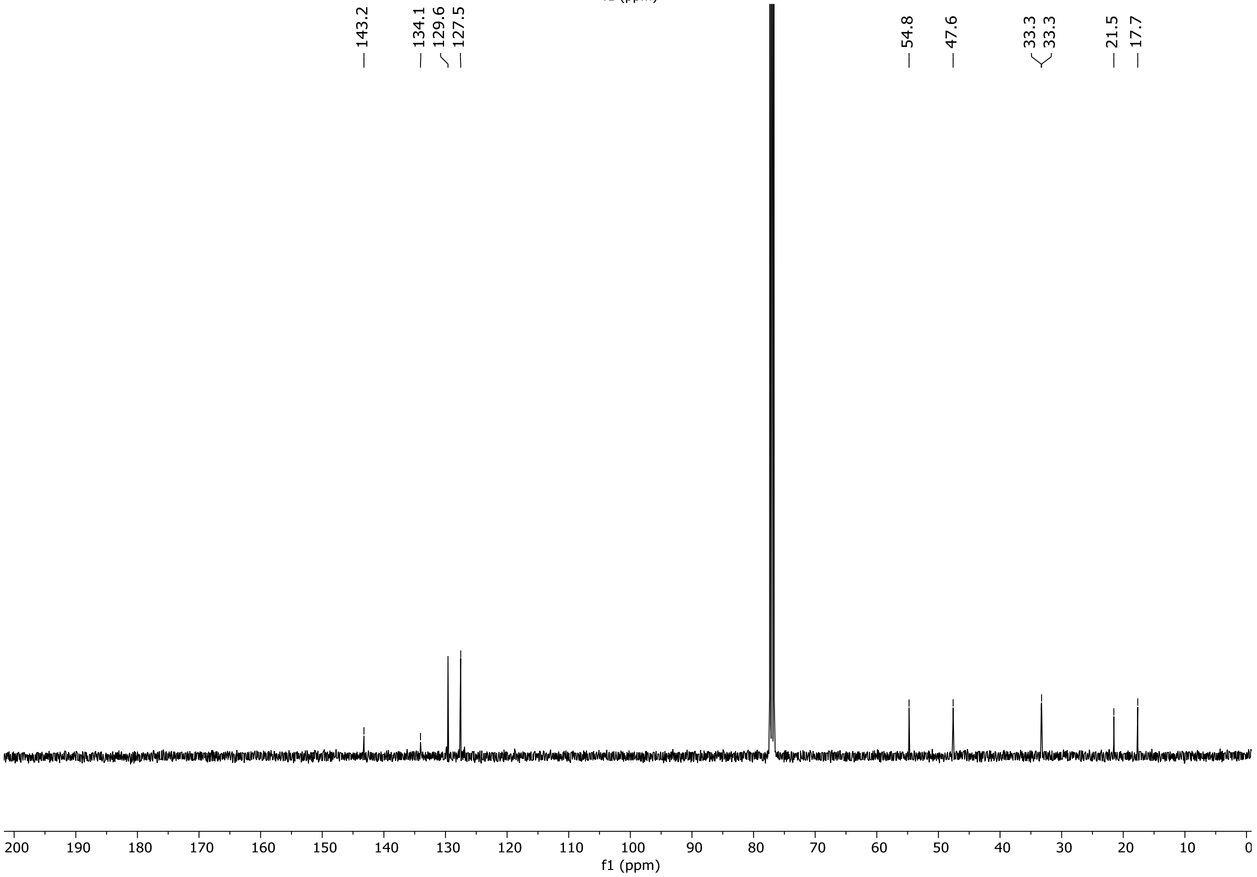
143.2
134.1
129.6
127.5
54.8
47.6
33.3
33.3
21.5
17.7
f1 (ppm)

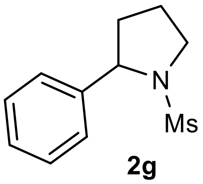
N
Ms

2g

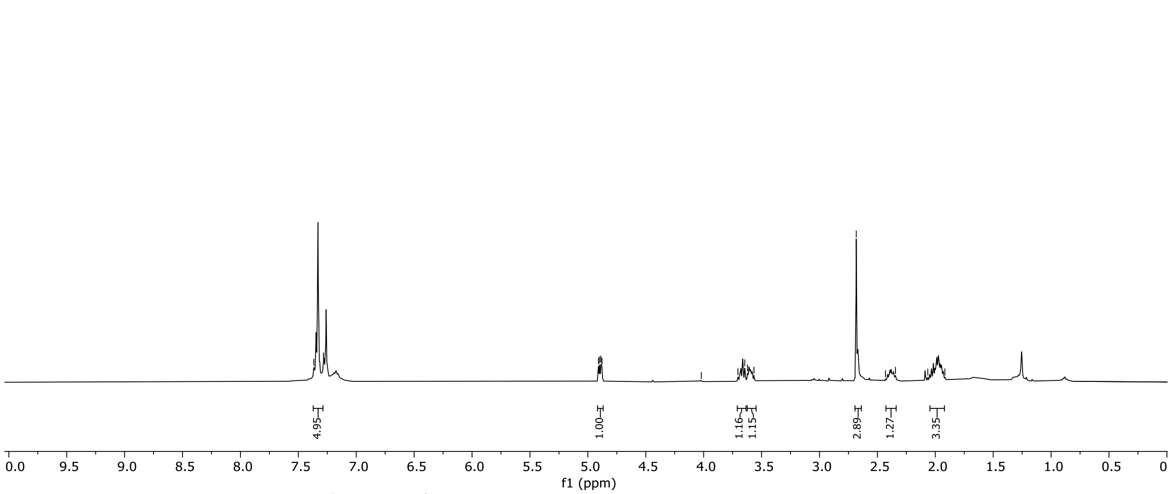
7.37
7.28
4.91
4.90
4.89
4.88
4.02
3.70
3.65
3.62
3.57
2.68
2.43
2.34
2.07
1.92
4.95
1.00
1.16
1.15
2.89
1.27
3.35
f1 (ppm)

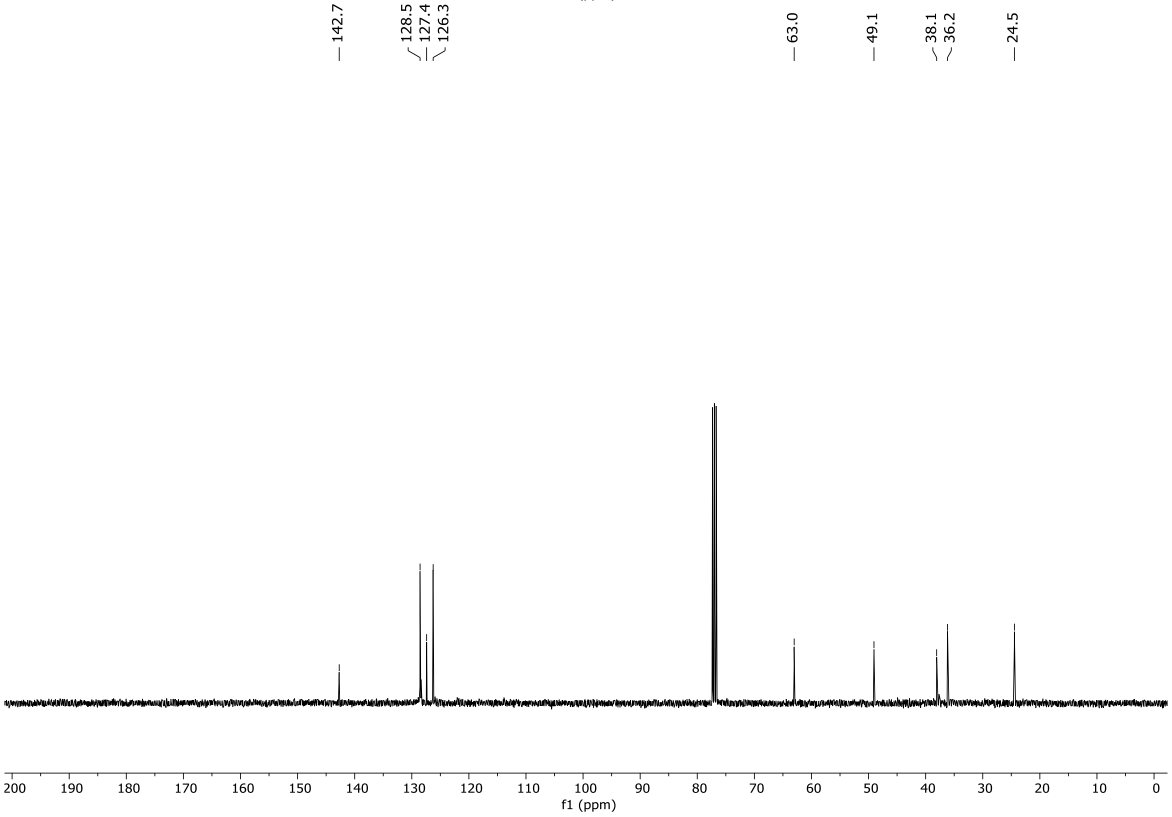
142.7
128.5
127.4
126.3
63.0
49.1
38.1
36.2
24.5
f1 (ppm)

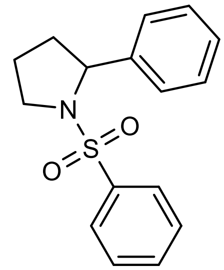
O
S
N
O

2h

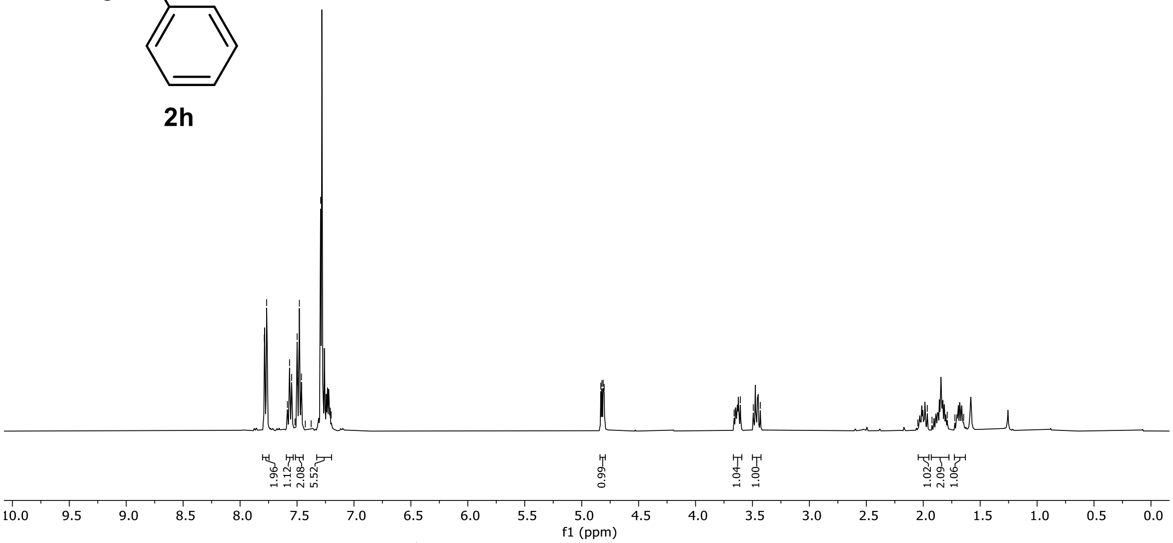
7.79
7.77
7.58
7.57
7.55
7.52
7.50
7.49
7.48
7.46
7.43
7.38
7.29
7.20
4.83
4.82
4.81
4.80
3.66
3.61
3.49
3.43
2.05
1.96
1.92
1.79
1.72
1.65
1.96
1.12
2.08
5.52
0.99
1.04
1.00
1.02
2.09
1.06
f1 (ppm)

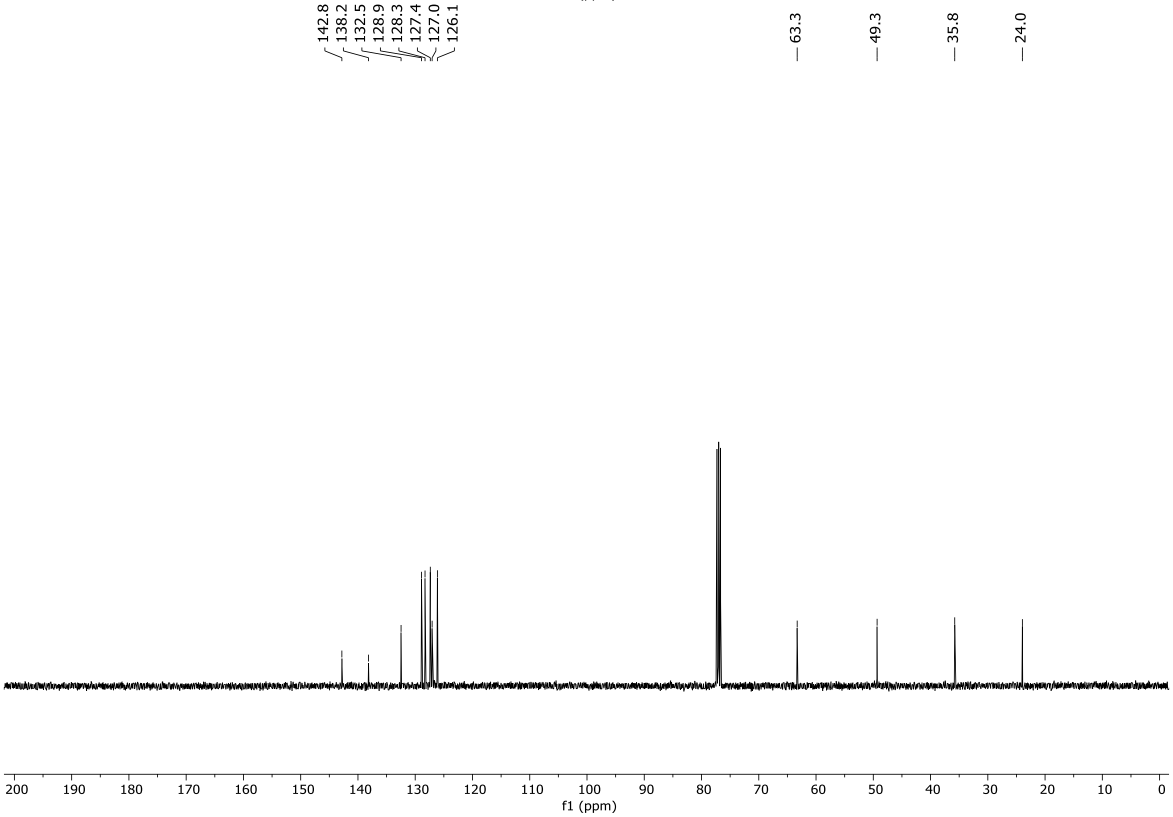
142.8
138.2
132.5
128.9
128.3
127.4
127.0
126.1
63.3
49.3
35.8
24.0
f1 (ppm)

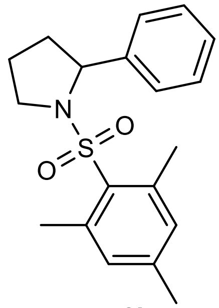
N
S
O
O

2i

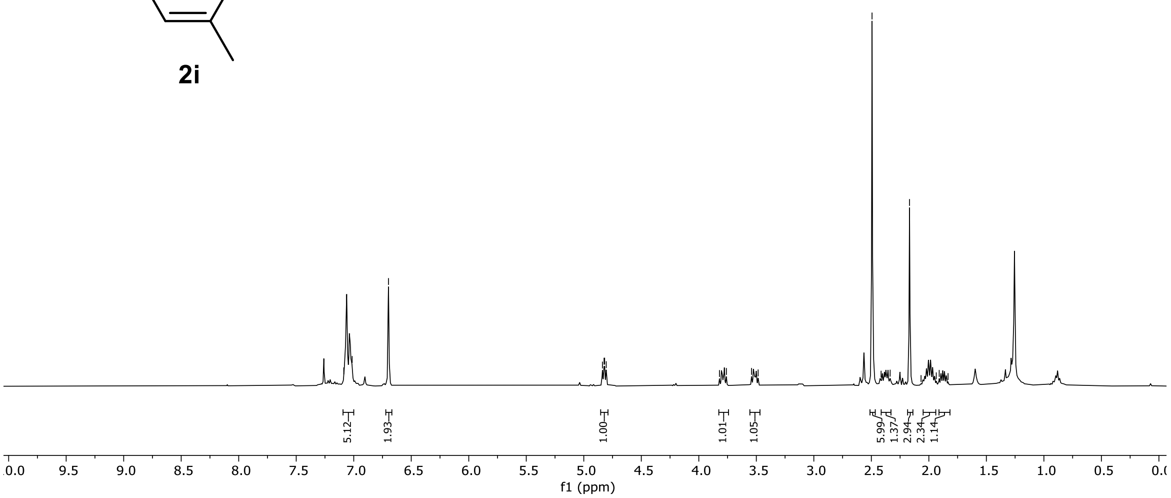
7.09
7.01
6.70
4.84
4.82
4.82
4.80
3.82
3.76
3.54
3.48
2.49
2.42
2.34
2.17
2.07
1.94
1.91
1.83
5.12
1.93
1.00
1.01
1.05
5.99
1.37
2.94
2.34
1.14
f1 (ppm)

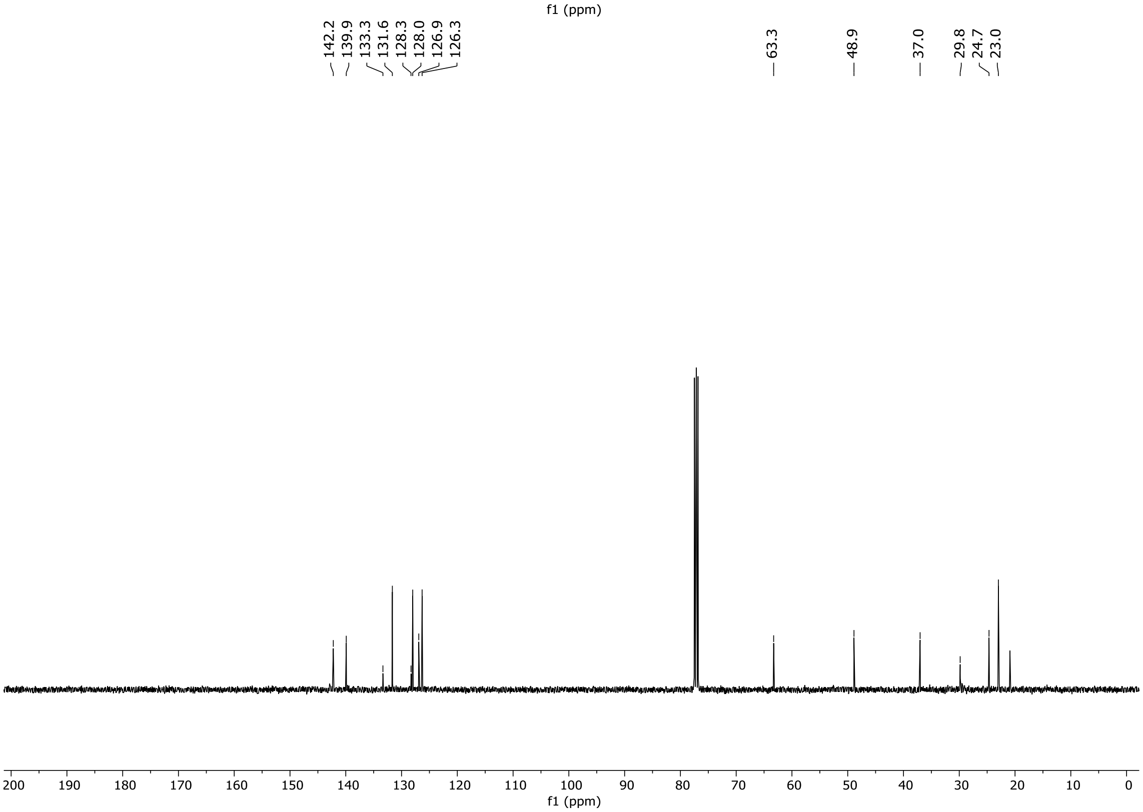
142.2
139.9
133.3
131.6
128.3
128.0
126.9
126.3
63.3
48.9
37.0
29.8
24.7
23.0
f1 (ppm)
